# A high-quality, long-read genome assembly of the endangered ring-tailed lemur (*Lemur catta*)

**DOI:** 10.1093/gigascience/giac026

**Published:** 2022-04-01

**Authors:** Marc Palmada-Flores, Joseph D Orkin, Bettina Haase, Jacquelyn Mountcastle, Mads F Bertelsen, Olivier Fedrigo, Lukas F K Kuderna, Erich D Jarvis, Tomas Marques-Bonet

**Affiliations:** Department of Medicine and Life Sciences (MELIS), Institut de Biologia Evolutiva, Universitat Pompeu Fabra-CSIC, Barcelona 08003, Spain; Department of Medicine and Life Sciences (MELIS), Institut de Biologia Evolutiva, Universitat Pompeu Fabra-CSIC, Barcelona 08003, Spain; Département d'anthropologie, Université de Montréal, Montréal, QC H3T 1N8, Canada; The Vertebrate Genomes Lab, The Rockefeller University, New York, NY 10065, USA; The Vertebrate Genomes Lab, The Rockefeller University, New York, NY 10065, USA; Department of Veterinary and Animal Sciences, Faculty of Health and Medical Sciences, University of Copenhagen, Frederiksberg C 1870, Denmark; Center for Zoo and Wild Animal Health, Copenhagen Zoo, Frederiksber 1870, Denmark; The Vertebrate Genomes Lab, The Rockefeller University, New York, NY 10065, USA; Department of Medicine and Life Sciences (MELIS), Institut de Biologia Evolutiva, Universitat Pompeu Fabra-CSIC, Barcelona 08003, Spain; The Vertebrate Genomes Lab, The Rockefeller University, New York, NY 10065, USA; Center for Zoo and Wild Animal Health, Copenhagen Zoo, Frederiksber 1870, Denmark; Howard Hughes Medical Institute, Chevy Chase, MD 20815, USA; Laboratory of Neurogenetics of Language, The Rockefeller University, NY 10065, USA; Department of Medicine and Life Sciences (MELIS), Institut de Biologia Evolutiva, Universitat Pompeu Fabra-CSIC, Barcelona 08003, Spain; Catalan Institution of Research and Advanced Studies (ICREA), Barcelona 08010, Spain; CNAG‐CRG, Centre for Genomic Regulation (CRG), Barcelona Institute of Science and Technology (BIST), Barcelon 08028a, Spain; Institut Català de Paleontologia Miquel Crusafont, Universitat Autònoma de Barcelona, Cerdanyola del Vallès 08193, Spain

**Keywords:** Strepsirrhine, primate, long-read assembly, ALUs, repeats, lemuridae, mitogenome, scaffolding

## Abstract

Background: The ring-tailed lemur (*Lemur catta*) is a charismatic strepsirrhine primate endemic to Madagascar. These lemurs are of particular interest, given their status as a flagship species and widespread publicity in the popular media. Unfortunately, a recent population decline has resulted in the census population decreasing to <2,500 individuals in the wild, and the species's classification as an endangered species by the IUCN. As is the case for most strepsirrhine primates, only a limited amount of genomic research has been conducted on *L. catta*, in part owing to the lack of genomic resources. Results: We generated a new high-quality reference genome assembly for *L. catta* (mLemCat1) that conforms to the standards of the Vertebrate Genomes Project. This new long-read assembly is composed of Pacific Biosciences continuous long reads (CLR data), Optical Mapping Bionano reads, Arima HiC data, and 10X linked reads. The contiguity and completeness of the assembly are extremely high, with scaffold and contig N50 values of 90.982 and 10.570 Mb, respectively. Additionally, when compared to other high-quality primate assemblies, *L. catta* has the lowest reported number of Alu elements, which results predominantly from a lack of AluS and AluY elements. Conclusions: mLemCat1 is an excellent genomic resource not only for the ring-tailed lemur community, but also for other members of the Lemuridae family, and is the first very long read assembly for a strepsirrhine.

## Context

The strepsirrhines are a remarkably diverse radiation of primates that includes more than one-quarter of all recognized primate species [1]. The vast majority of strepsirrhines (103 species) are members of the Lemuroidea, colloquially known as “lemurs,” and endemic to Madagascar. Despite their geographic isolation, the lemur radiation is exceptionally diverse, including both the smallest living primate (*Microcebus berthae*) and one of the largest (the recently extinct subfossil lemur, *Archaeoindris fontoynontyii*) [2, 3]. Although lemurs are highly diverse, they are comparatively understudied relative to other primates, and ∼87% of species are threatened with extinction, raising major conservation challenges [1].

**Figure 1:**
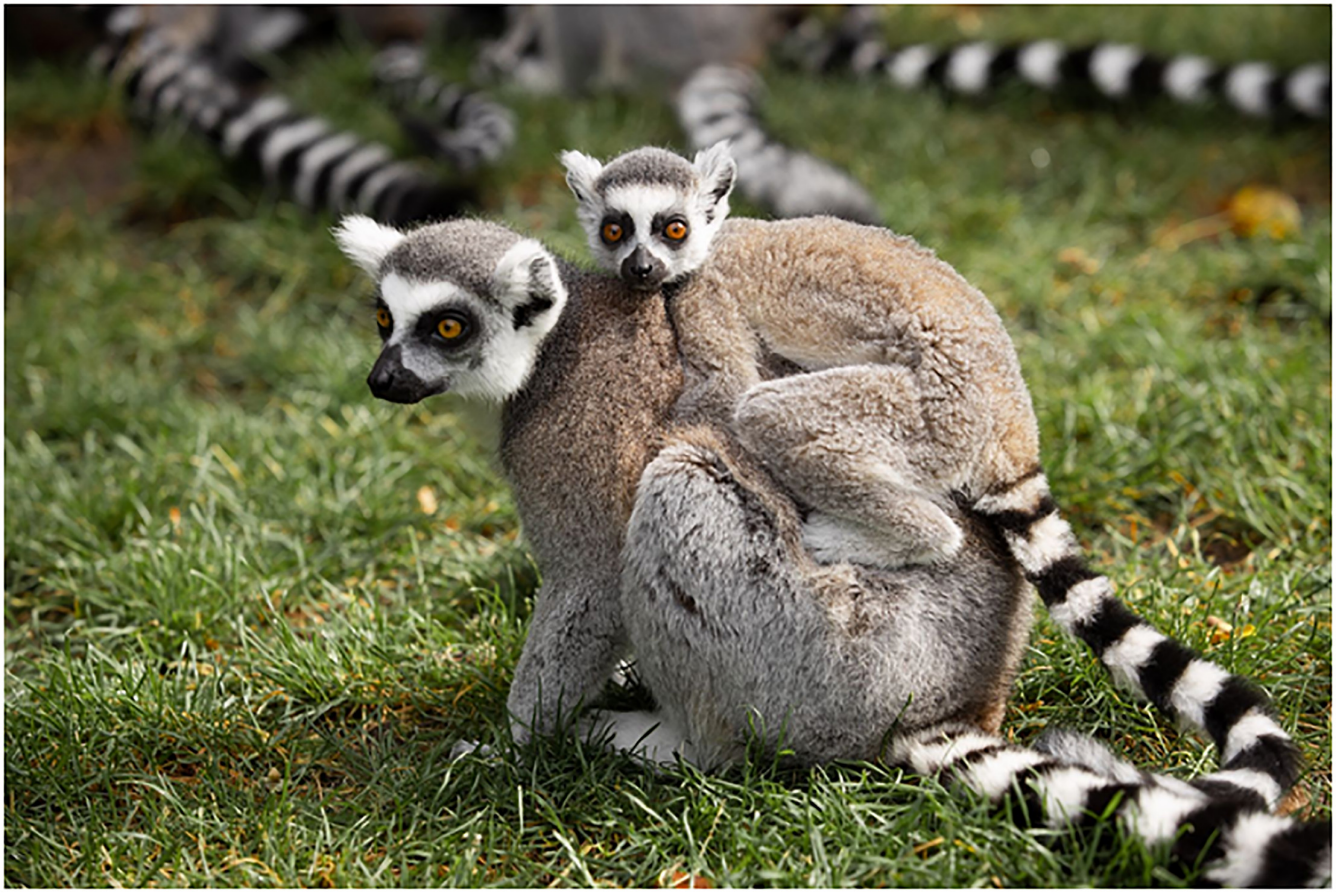
Ring-tailed lemur (*L. catta*); photo courtesy of Copenhagen Zoo.

Of particular interest, both ecologically and in the public imagination, are ring-tailed lemurs (*Lemur catta*, NCBI:txid9447; Fig. 1). Ring-tailed lemurs are medium-bodied, ecologically flexible members of the Lemuridae family and the sole member of the genus *Lemur* [ 4–6]. In contrast to most other Lemuridae, *L. catta* predominantly inhabit the dry and seasonal forests of southern Madagascar [7]. They consume an omnivorous diet mostly of fruit and leaves and engage in a multi-male multi-female social structure with a polygynandrous mating system [7]. Ring-tailed lemurs are under severe conservation pressure; they are classified as "endangered" by the International Union for Conservation of Nature (IUCN) [8], resulting primarily from deforestation, hunting, and capture for the pet trade. A recent population census has revealed a dramatic population decline, with as few as 2,200 individuals remaining in the wild [9]. Of further concern, the species is distributed across a highly fragmented range with only 8 populations of ≥100 individuals remaining [9]. Despite this near-term population decline, a recent microsatellite analysis indicates that the genetic diversity of *L. catta* populations could be exceptionally high, with evidence of genetic isolation by distance throughout their geographic range [6].

From a genomic perspective, relatively little is known about ring-tailed lemurs (and strepsirrhines more broadly). Genome assemblies have been published for 18 strepsirrhine species, but none of these assemblies has a contig N50 value >1 Mb, and only 3 of them are >100 kb [10]. Recently, an *L. catta* genome (LemCat_v1_BIUU) was assembled by the Zoonomia consortium [11]; given that it is derived from Illumina short reads, its metrics and application are still limited compared to the genome quality of recent highly contiguous assemblies [12]. This general lack of genomic resources remains a limitation for the comparative and population genomics of lemurs.

Here, we present a new high-quality genome assembly of *L. catta* (mLemCat1) that conforms to the standards of the Vertebrate Genomes Project (VGP). mLemCat1 was assembled with a combination of Pacific Biosciences (PacBio) continuous long reads (CLR data), Optical Mapping Bionano reads, Arima HiC data, and 10X linked-reads. Our new assembly will allow for a deep assessment of the genome biology and conservation genomics of endangered ring-tailed lemurs. Additionally, given the paucity of high-contiguity strepsirrhine assemblies, it will allow major advances in the genomics across the Lemuridae family.

## Data Description

Bionano: San Diego/CA/USA; Qubit: Thermo Fisher Scientific: Waltham/MA/USA; PacBio: Menlo Park/CA/USA; Sage Science: Beverly/MA/USA; 10xgenomics: Pleasanton/CA/USA; Illumina: San Diego/CA/USA; Arima Genomics: San Diego/CA/USA; KAPA (Roche) Basel Switzerland.

### Library preparation and sequencing

A sample of spleen tissue was collected post-mortem from a male at the Copenhagen Zoo (Denmark) in 2015 and immediately flash-frozen (ZIMS Global Accession No. GAN: DKL15-03 323). We isolated 30 μg of ultra-high molecular weight DNA (uHMW) from 35 mg of flash-frozen spleen tissue using the agarose plug Bionano Genomics protocol for animal tissue (DNA isolation fibrous tissue protocol No. 30071C). uHMW DNA quality was assessed by a Pulsed-Field Gel assay and quantified with a Qubit 2 Fluorometer (Supplementary Fig. S1).

A 10 µg quantity of uHMW DNA was sheared using a 26G blunt-end needle (PacBio protocol PN 101–181-000 Version 05). A large-insert PacBio library was prepared using the Pacific Biosciences Express Template Prep Kit v2.0 (No. 100–938-900) following the manufacturer protocol. The library was then size selected (>20 kb) using the Sage Science BluePippin Size-Selection System. A total of 23 PacBio 1M v3 (#101–531-000) smrtcells were sequenced on the Sequel instrument (PacBio Sequel System, RRID:SCR_017989) (sequencing kit 3.0 No. 101–597-800) with a 10-hour movie and 2 hours pre-extension time. Unfragmented uHMW DNA was used to generate a linked-reads library on the 10X Genomics Chromium (Genome Library Kit & Gel Bead Kit v2 PN-120258, Genome Chip Kit v2 PN-120257, i7 Multiplex Kit PN-120262). This 10X library was sequenced on an Illumina Novaseq (Illumina NovaSeq 6000 Sequencing System, RRID:SCR_016387) S4 150-bp PE lane. uHMW DNA was labeled for Bionano Genomics optical mapping (BioNano Irys system, RRID:SCR_016754) using the Bionano Prep Direct Label and Stain (DLS) Protocol (30206E) and run on 1 Saphyr (Saphyr, RRID:SCR_017992) instrument chip flow cell. Hi-C preparation was performed by Arima Genomics using the Arima-HiC kit (P/N: A510008) and an Illumina-compatible library was generated using the KAPA Hyper Prep kit (P/N: KK8504). This library was then sequenced on an Illumina HiSeq X Ten (Illumina HiSeq X Ten, RRID:SCR_016385) (150 bp PE) at ∼60× coverage following the manufacturer's protocols. Assuming a genome size of 3.21 Gb from the GoaT database [13], the present genome (mLemCat1) has been produced with 86.43× of 10X linked-reads data, 66.68× of Arima data, 154.57× of Bionano data, and 62.88× of PacBio data.

### *De novo* assembly

The genome was assembled following the VGP standard pipeline v1.6 [12], and the specific parameter settings are available on the VGP GitHub repository (Additional File 1). Specifically, contigs were generated using FALCON  (FALCON, RRID:SCR_018804) [14] and FALCON-Unzip [15], producing primary and alternate assemblies. We used purge_dups (purge dups, RRID:SCR_021173) [16] to identify false duplications caused by regions of high heterozygosity. Purged contigs were removed from the primary assembly and added to the alternate assembly. We then scaffolded the primary assembly using 10X linked-read data with Scaff10X V2.0 [17], Bionano optical maps with Bionano Solve V.2.1 [18], and Arima Hi-C data with Salsa V2.2 [19]. We assembled the mitochondrial genome separately using MitoVGP [20] with PacBio and 10X data. The primary scaffolds, alternate contigs, and mitochondrial assembly were polished simultaneously. We first performed Polishing and gap filling with the original PacBio data using Arrow [14], followed by 2 rounds of short-read polishing using the 10X linked-read data. Specifically, 10X data were mapped to the assembly using Longranger V2.1.3 [21] and polishing was done with FreeBayes (FreeBayes, RRID:SCR_010761) [22]. All computing was performed on the DNAnexus (DNAnexus, RRID:SCR_011884) cloud platform.

### Genome quality assessment

Compared to the currently available short-read*L. catta* genome (LemCat_v1_BIUU) [11], the new mLemCat1 assembly has higher contiguity values, fewer scaffolds, and a slightly smaller assembly size (Table 1). We generated basic continuity assembly metrics for both assemblies using QUAST V5.0.2 (QUAST, RRID:SCR_001228) [23], which are presented in Table 1. The assembly has a total scaffold size of 2.122 Gb within 141 scaffolds. The mLemCat1 contig and scaffold N50 values are 10.570 and 90.982 Mb, representing 20.41- and 421.21-fold increases, respectively, compared with the LemCat_v1_BIUU assembly. In comparison with the human genome assembly (hg38), the L95 and N95 statistics (L95 = 24; N95 = 46.710 Mb for hg38; L95 = 28; N95 = 21.924 Mb for mLemCat1) are similar, given the expected chromosomes for both (22 autosomes + 2 sexual chromosomes in human, and 27 autosomal + 2 sex chromosomes for *L. catta*) [24]. Further comparison can be found in Supplementary Table S1. The overall GC content of this assembly is 40.48%.

**Table 1:** Genome quality metrics for the mLemCat1 genome assembly compared to previous assembly and standards

Quality category	Quality metric	VGP standard	mLemCat1	LemCat_v1_BIUU
**Continuity**	No. scaffolds		141	575,427
	Scaffold N50 (Mb)	23–480	90.982	0.216
	Largest scaffold (Mb)		285.823	2.320
	No. contigs		518	580,026
	Contig N50 (Mb)	1–25	10.570	0.158
	Largest contig (Mb)		40.360	1.312
	Gaps/Gb	75–1,500	179.5	2,001.3
	Span (Gb)		2.122	2.298
**Structural accuracy**	False duplications (%)	0.2–5.0	0.39	
**Base accuracy**	Base pair QV	39–43	44.45	
	*k*-mer completeness (%)	87–98	91.45	
**Functional completeness**	Genes (BUSCOs [S]) (%)	82–98	93.8	84.6
**Chromosome status**	Organelles^a^	1 Complete allele	1 Complete allele	

No.: Number of. S: single-copy genes. BUSCOs database is vertebrata_odb10 (n=3,354) and the BUSCO version is: V4.0.6.

a For example, mitochondrial.

The mLemCat1 assembly has a high level of accuracy and completeness that conforms to the proposed standards of the VGP [12]. We assessed the base and structural accuracies of the assembly with Merqury V1.1, using a Meryl V1.7 database [25] based on 130.708 Gb (84× coverage) of 10X linked-reads. The base pair QV of the primary assembly is 44.35, which exceeds the VGP standard. The *k*-mer completeness is 91.45%. We classified the structural accuracy using the false duplications percentage calculated in the false_duplications.sh script from Merqury V1.1. The assembly is estimated to have 0.39% false duplications based on the percentage of *k*-mers found in unexpected copy numbers.

**Figure 2:**
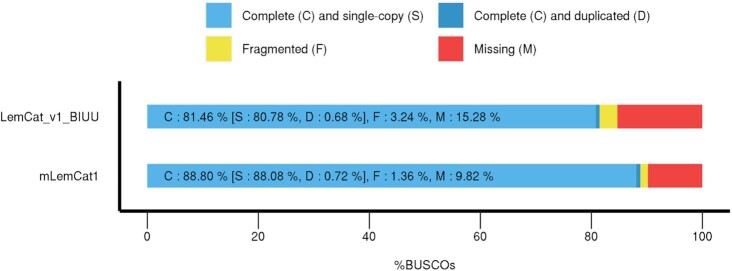
BUSCO assessment results. Comparison between mLemCat1 and LemCat_v1_BIUU *Lemur catta* assemblies using the Primates_ODB10 database (n = 13,780). The new mLemCat1 assembly shows a 7.3% increase in complete single-copy orthologous genes.

To assess the functional completeness of the assembly, we recovered BUSCO genes from both mLemCat1 and the existing Illumina-based assembly (LemCat_v1_BIUU) (Fig. 2). Specifically, we conducted a gene completeness assessment using BUSCO (BUSCO, RRID:SCR_015008) V4.0.6 [26], setting human as the reference species in the –augustus_species parameter, and using the primates_OrthoDB10 database, which comprises a total of 13,780 genes. Of the 13,780 possible BUSCOs, we identified 12,138 single-copy (88.1%), 100 duplicates (0.7%), and 188 fragmented genes (1.4%) in mLemCat1, leaving 9.8% of BUSCOs missing. In contrast, we could only recover 11,132 single-copy BUSCOs (80.8%) from LemCat_v1_BIUU, with 15.3% of BUSCO genes missing. We also ran the same analyses, but changed the database to vertebrata_odb10 and obtained even better results for mLemCat1 compared to LemCat_v1_BIUU, which we report in Table 1. The present assembly (mLemCat1) can be useful to create synteny plots between the present species and others, such as humans (Supplementary Fig. S2) because it has N50 statistics comparable to other high-quality primate genomes, like the pig-tailed macaque [27], olive baboon [28], and golden snub-nosed monkey [29] genome assemblies that have been recently published (Supplementary Table S2).

### Mitogenome of *L. catta*

We assembled a gapless mitochondrial genome with a span of 17,086 bp using both PacBio CLR (long reads) and 10X data (short reads) using MitoVGP V2.2 with additional parameters “-f 18 000 -v LENIENT”, as described in the Additional File 2: Table S3 of Formenti et al. [20], and annotated the assembly using the MITOS2 web server [30]. With the annotation results we plotted a map of the mitochondrion with GenomeVx [31] (Supplementary Fig. S3). Thirteen main protein-coding genes have been annotated in this new mitogenome including nad1, nad2, nad3, nad4, nad4L, nad5, nad6, cox1, cox2, cox3, atp6, atp8, and cob.

### Analysis of the repeatome

**Figure 3:**
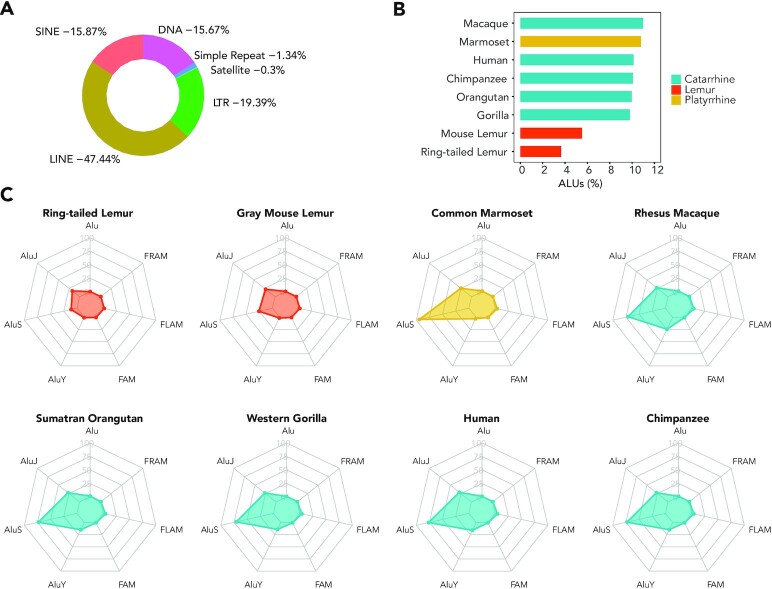
(A) Percentages of elements in the *L. catta* genome (mLemCat1) masked by RepeatMasker. (B) Percentage of Alus masked in primate long-read assemblies. (C) Spider plots of the total number of different Alu-like elements masked in each genome assembly. Lemurs have fewer AluS elements than anthropoid primates. Axis values represent 1,000× events: FAM (Fossil Alu Monomer); FLAM (Free Left Alu Monomers; FRAM (Free Right Alu Monomer; AluJ (oldest); AluS (intermediate); AluY (youngest); Alu (non-specified). LINE: long interspersed nuclear element; LTR: long terminal repeat; SINE: short interspersed nuclear element [31].

To assess the structure and variety of repeat elements in the *L. catta* genome, we analyzed mLemCat1 with RepeatMasker  (RepeatMasker, RRID:SCR_012954) V4.1.2-p1. Non-default settings included the use of sensitive mode, the query assumed species set to primates, nhmmscan 3.3.2 (Nov 2020), and FamDB: HMM-Dfam_3.3, without the exclusion of simple repeats. In total, 50.32% of the bases in the *L. catta* genome (mLemCat1) are masked as interspersed repeats, including long and short interspersed nuclear elements, long terminal repeats, and DNA elements (Fig. 3A, Supplementary Table S3). In general terms, the portion of the genome that comprises repetitive elements is similar to that reported for other high-quality catarrhine genomes [32,33], although there are fewer satellites (0.30%), simple repeats (0.68%), and low-complexity elements (0.13%) (Supplementary Table S3).

**Figure 4:**
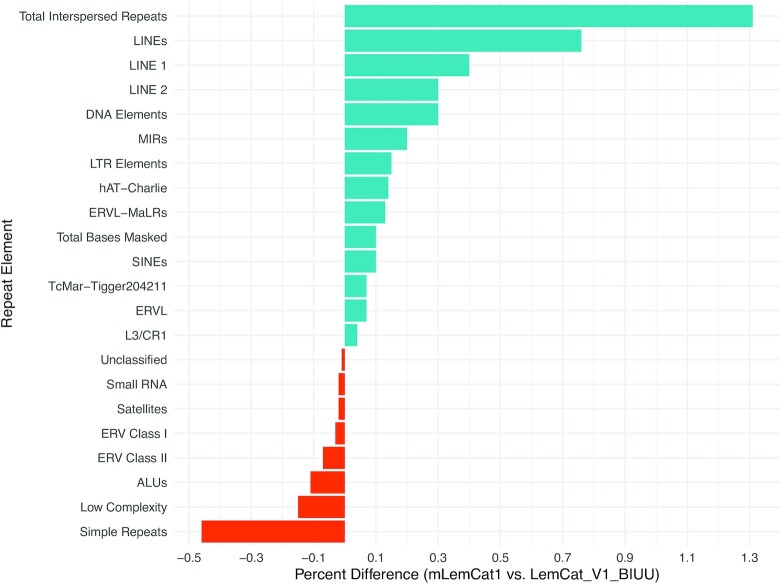
Comparison of repeat variety and structure between mLemCat1 and LemCat_V1_BIUU assemblies. LINE: long interspersed nuclear element; LTR: long terminal repeat; SINE: short interspersed nuclear element.

In comparison with the previous Illumina-only assembly (LemCat_v1_BIUU) we observed minor differences in the structure and variety of repeat elements (Fig. 4). The new long-read–based assembly has 1.31% more interspersed repeats (50.32% vs 49.01%), and a higher percentage of sequence in each repeat subtype, except for satellites, simple repeats, low-complexity elements, and ERV classes I and II. We also observed both a lower percentage of sequence and a smaller number of Alu events in mLemCat1. Additionally, the total number of masked bases is lower in the new assembly, but they represent a higher percentage of the sequence, owing to mLemCat1 having a shorter span.

Alus are the most abundant repeat elements in the human genome, and differences in their rates, distribution, and proliferation could have led to distinct functional changes in multiple primate lineages [34]. Alu elements have been present since the earliest stages of primate evolution, are frequently located in gene-rich regions, and may play an important role in gene regulation [35–38]. To compare the Alu repeat landscape of *L. catta* with those of other highly contiguous primate assemblies, we ran the same version of RepeatMasker as above adding the -alu option. The genomes used for the comparison were long-read–based assemblies, including human (hg38), chimpanzee (panTro6), western gorilla (gorGor6), Sumatran orangutan (ponAbe3), rhesus macaque (rheMac10), common marmoset (calJac4), and gray mouse lemur (Mmur_3.0) (Supplementary Table S4).

We identified substantially fewer Alu elements in the lemur genomes (*L. catta* and *Microcebus murinus*) than those of the catarrhines, with the fewest being found in the *L. catta* genome (3.66% of repeat elements) (Fig. 3B, Supplementary Table S5). In contrast to the other primates assessed, for which AluS elements are most abundant, AluJ is the most common element in mLemCat1 (54.17% of Alu events). Both lemurs have fewer AluS events than the anthropoids and fewer AluY events than the catarrhines, consistent with previous reports of the expansion of these 2 families after the Catarrhini-Strepsirrhini split [39]. The fact that the common marmoset has the highest number of AluS elements (Fig. 3C) confirms that the burst that started before the Catarrhini and Platyrrhini parvorders diverged continued with different activity in both lineages after their split. Recent Alu activity (AluY events) is most abundant in catarrhines, particularly the rhesus macaque, which, when compared to great apes (Fig. 3C), has a higher overall percentage of Alus (Fig. 3B).

## Conclusion

We have assembled a new high-quality genome reference for the ring-tailed lemur (*L. catta*) that satisfies the VGP quality assembly standards. Compared to pre-existing genomic resources, the new assembly has higher contiguity and completeness and contains more single-copy complete BUSCO genes with fewer fragmented or missing genes. Additionally, we analyzed the *L. catta* repeatome and observed substantially fewer Alu events compared to other high-quality primate assemblies. This assembly illustrates how long-reads and further scaffolding data such as HiC or optical mappings can drastically improve the contiguity and completeness of an assembly, which also allows for improved analysis of structural variation. We suggest that this new assembly will be an excellent resource for the mammalian genomics community, with particular value for the conservation genomics of lemurs (Fig. 1 ).

## Data Availability

The raw sequencing data and assembly are available via NCBI BioProject: PRJNA562215. mLemCat1 assembly and the raw reads used to generate it can be accessed at GenomeArk [41]. The complete mitogenome of mLemCat1 is available in Genomeark as mLemCat1.MT.20190820.fasta.gz [41]. Specific command line parameters are available in Additional File 1. The supporting datasets are available in the *GigaScience* database (GigaDB) [40].

## Additional Files

**Additional File 1:** Links to the websites with the assembly pipeline specifics used to create *Lemur catta* (mLemCat1) genome assembly and command lines used to perform the different analyses.

**Supplementary Figure S1:** Pulse Field Gel assay (Sage Pippin Pulse) used for quality control of the ultra-High Molecular Weight DNA

**Supplementary Figure S2:** A chromosomal overall synteny plot between Lemur catta (mLemCat1 assembly; vertical axis) and Homo sapiens (hg38 assembly; horizontal axis)

**Supplementary Figure S3:** Representation of the Lemur catta mitogenome (mLemCat1.MT.20190820)

**Supplementary Table S1:** Continuity metrics of human (hg38) and Lemur catta (mLemCat1) assemblies

**Supplementary Table S2:** Comparison of scaffold N50 and assembly size of the latest primate genomes published in GigaScience

**Supplementary Table S3:** Repetitive elements identified by RepeatMasker in the L. catta genome assembly (mLemCat1)

**Supplementary Table S4:** Primate assembly lengths and respective quantifications of their Alu content

**Supplementary Table S5:** Alu-monomers and Alu subfamilies count in each assembly

giac026_GIGA-D-21-00335_Original_Submission

giac026_GIGA-D-21-00335_Revision_1

giac026_Response_to_Reviewer_Comments_Revision_1

giac026_Reviewer_1_Report_Original_Submission Xiao-Guang Qi -- 11/15/2021 Reviewed

giac026_Reviewer_2_Report_Original_Submission Morteza Roodgar -- 11/29/2021 Reviewed

giac026_Reviewer_2_Report_Revision_1 Morteza Roodgar -- 2/8/2022 Reviewed

giac026_Supplemental_Files

## Abbreviations

bp: base pairs; BUSCO: Benchmarking Universal Single-Copy Orthologs; Gb: gigabase pairs; IUCN: International Union for Conservation of Nature; kb: kilobase pairs; Mb: megabase pairs; NCBI: National Center for Biotechnology Information; PacBio: Pacific Biosciences; SMRT: single-molecule real-time; uHMW: ultra-high molecular weight; VGP: Vertebrate Genomes Project.

## Competing Interests

“LFKK is currently an employee of Illumina Inc.” All other authors declare no competing interests.

## Funding

The project that gave rise to these results received the support of 2 fellowships from "la Caixa" Foundation (ID 100010434) and from the European Union's Horizon 2020 research and innovation programme under the Marie Skłodowska-Curie grant agreement No. 847648. The fellowship codes are LCF/BQ/PI20/11760004 and LCF/BQ/DR20/11790032. T.M.B. is supported by funding from the European Research Council (ERC) under the European Union's Horizon 2020 research and innovation programme (grant agreement No. 864203), BFU2017-86471-P (MINECO/FEDER, UE), “Unidad de Excelencia María de Maeztu,” funded by the AEI (CEX2018-000792-M), Howard Hughes International Early Career, NIH 1R01HG010898-01A1, and Secretaria d'Universitats i Recerca and CERCA Programme del Departament d'Economia i Coneixement de la Generalitat de Catalunya (GRC 2017 SGR 880).

## Authors' Contributions

M.P.F. and J.D.O. analyzed the data; J.M. and B.H. generated the data; B.H. generated the draft assembly; M.F.B. collected the samples. M.P.F. and J.D.O. wrote the manuscript with contributions from all authors. T.M.B., E.D.J., O.F., and L.F.K.K. designed the research.
