# Supplementary material for: A high-quality, long-read genome assembly of the endangered ring-tailed lemur (Lemur catta)
Source: Gigascience. 2022 Apr 1;11:giac026. doi: 10.1093/gigascience/giac026 (PMC8975718; doi:10.1093/gigascience/giac026)
Supplement: giac026_GIGA-D-21-00335_Original_Submission [file giac026_giga-d-21-00335_original_submission.pdf]

GigaScience

Data Note: A high-quality, long-read genome assembly of the endangered ring-tailed lemur (Lemur catta)

--Manuscript Draft--

**Manuscript Number:**	GIGA-D-21-00335
**Full Title:**	Data Note: A high-quality, long-read genome assembly of the endangered ring-tailed lemur (Lemur catta)
**Article Type:**	Data Note
**Funding Information:**	Departament d'Economia i Coneixement de la Generalitat de Catalunya (GRC 2017 SGR 880) — Dr. Tomas Marques-Bonet<br>"la Caixa" Foundation (LCF/BQ/PI20/11760004) — Dr. Joseph D. Orkin<br>"la Caixa" Foundation (LCF/BQ/DR20/11790032) — MSc Marc Palmada-Flores<br>H2020 European Research Council (864203) — Dr. Tomas Marques-Bonet<br>H2020 Marie Skłodowska-Curie Actions (847648) — Dr. Joseph D. Orkin<br>"Unidad de Excelencia María de Maeztu" (CEX2018-000792-M) — Dr. Tomas Marques-Bonet<br>Howard Hughes Medical Institute (NIH 1R01HG010898-01A1) — Dr. Tomas Marques-Bonet

Abstract: The ring-tailed lemur (Lemur catta) is a charismatic strepsirrhine primate endemic to the island of Madagascar. These lemurs are of particular interest, given their status as a flagship species and widespread publicility in popular media. Unfortunately, a recent population decline has caused the census population to fall below 2500 individuals in the wild, and their classification as an endangered species by the IUCN. As is the case for most strepsirrhine primates, only a limited amount of genomic research has been conducted on L. catta , in part due to the lack of genomic resources. We generated a new high-quality reference genome assembly for L. catta (mLemCat1) that conforms to the standards of the Vertebrate Genomes Project. This new long-read assembly is composed of PacBio continuous long reads (CLR reads), Optical Mapping Bionano reads, Arima HiC data, and 10X linked-reads. The contiguity and completeness of the assembly is extremely high, with scaffold and contig N50 values of 90.982 Mbp and 10.570 Mbp, respectively. Additionally, when compared to other high-quality primate assemblies, L. catta has the lowest reported number of Alu elements, which results predominantly from a lack of AluS and AluY elements. mLemCat1 is an excellent genomic resource not only for the ring-tailed lemur community, but also for other members of the Lemuridae family and is the first very long read assembly for a strepsirrhine.

**Corresponding Author:**	Marc Palmada-Flores<br>Institute of Evolutionary Biology: Instituto de Biologia Evolutiva<br>Barcelona, SPAIN
**Corresponding Author Secondary Information:**	
**Corresponding Author's Institution:**	Institute of Evolutionary Biology: Instituto de Biologia Evolutiva
**Corresponding Author's Secondary Institution:**	
**First Author:**	Marc Palmada-Flores
**First Author Secondary Information:**	
**Order of Authors:**	Marc Palmada-Flores
	Joseph D. Orkin
	Bettina Haase

Powered by Editorial Manager® and ProduXion Manager® from Aries Systems Corporation

	Jacquelyn Mountcastle
	Mads Frost-Bertelsen
	Olivier Fedrigo
	Lukas Kuderna
	Erich D. Jarvis
	Tomas Marques-Bonet
**Order of Authors Secondary Information:**	
**Additional Information:**	
**Question**	**Response**
Are you submitting this manuscript to a special series or article collection?	No
**Experimental design and statistics**<br><br>Full details of the experimental design and statistical methods used should be given in the Methods section, as detailed in our Minimum Standards Reporting Checklist. Information essential to interpreting the data presented should be made available in the figure legends.<br><br>Have you included all the information requested in your manuscript?	Yes
**Resources**<br><br>A description of all resources used, including antibodies, cell lines, animals and software tools, with enough information to allow them to be uniquely identified, should be included in the Methods section. Authors are strongly encouraged to cite Research Resource Identifiers (RRIDs) for antibodies, model organisms and tools, where possible.<br><br>Have you included the information requested as detailed in our Minimum Standards Reporting Checklist?	Yes
**Availability of data and materials**<br><br>All datasets and code on which the	Yes

conclusions of the paper rely must be either included in your submission or deposited in publicly available repositories (where available and ethically appropriate), referencing such data using a unique identifier in the references and in the “Availability of Data and Materials” section of your manuscript.<br><br>Have you have met the above requirement as detailed in our Minimum Standards Reporting Checklist?	

Powered by Editorial Manager® and ProduXion Manager® from Aries Systems Corporation

Manuscript

Click here to access/download;Manuscript;LemurCattaRef_MANUSCRIPT.do

Data Note: A high-quality, long-read genome assembly of the endangered ring-tailed lemur (*Lemur catta*)

Marc Palmada-Flores[1,*], Joseph D. Orkin[1,2*], Bettina Haase[3], Jacquelyn Mountcastle[3], Mads Frost-Bertelsen[4,5], Olivier Fedrigo[3], Lukas Kuderna[1], Erich D. Jarvis[3,6,7], Tomas Marques-Bonet[1,8,9,10,x]

1 Institut de Biologia Evolutiva, Universitat Pompeu Fabra-CSIC, Barcelona, Spain

2 Département d'anthropologie, Université de Montréal, 3150 Jean-Brillant, Montréal, Quebéc, Canada

3 The Vertebrate Genomes Lab, The Rockefeller University, New York, New York, USA

4 Department of Veterinary and Animal Sciences, Faculty of Health and Medical Sciences, University of Copenhagen, Frederiksberg C, Denmark

5 Center for Zoo and Wild Animal Health, Copenhagen Zoo, Frederiksberg, Denmark

6 Howard Hughes Medical Institute, Chevy Chase, Maryland, USA

7 Laboratory of Neurogenetics of Language, The Rockefeller University, New York, United States

8 Catalan Institution of Research and Advanced Studies (ICREA), Barcelona, Spain

9 CNAG- CRG, Centre for Genomic Regulation (CRG), Barcelona Institute of Science and Technology (BIST), Barcelona, Spain

10 Institut Català de Paleontologia Miquel Crusafont, Universitat Autònoma de Barcelona, Barcelona, Spain

* These authors contributed equally

x Corresponding Author

Abstract

The ring-tailed lemur (*Lemur catta*) is a charismatic strepsirrhine primate endemic to the island of Madagascar. These lemurs are of particular interest, given their status as a flagship species and widespread publicility in popular media. Unfortunately, a recent population decline has caused the census population to fall below 2500 individuals in the wild, and their classification as an endangered species by the IUCN. As is the case for most strepsirrhine primates, only a limited amount of genomic research has been conducted on *L. catta*, in part due to the lack of genomic resources. We generated a new high-quality reference genome assembly for *L. catta* (mLemCat1) that conforms to the standards of the Vertebrate Genomes Project. This new long-read assembly is composed of PacBio continuous long reads (CLR reads), Optical Mapping Bionano reads, Arima HiC data, and 10X linked-reads. The contiguity and completeness of the assembly is extremely high, with scaffold and contig N50 values of 90.982 Mbp and 10.570 Mbp, respectively. Additionally, when compared to other high-quality primate assemblies, *L. catta* has the lowest reported number of Alu elements, which results predominantly from a lack of AluS and AluY elements. mLemCat1 is an excellent genomic resource not only for the ring-

tailed lemur community, but also for other members of the Lemuridae family and is the first very long read assembly for a strepsirrhine.

Context:

The strepsirrhines are a remarkably diverse radiation of primates that includes more than one quarter of all recognized primate species [1]. The vast majority of strepsirrhines (103 species) are members of the Lemuroidea, colloquially known as “lemurs”, and endemic to Madagascar. Despite their geographic isolation, the lemur radiation is exceptionally diverse, including both the smallest living primate (*Microcebus berthae*) and one of the largest (the recently extinct subfossil lemur, *Archaeoindris fontoynontyii*) [2,3]. Although lemurs are highly diverse, they are remarkably understudied relative to other primates, and ~87% of species are threatened with extinction, raising major conservation challenges [1].

Of particular interest, both ecologically and in the public imagination, are ring-tailed lemurs (*Lemur catta*). Ring-tailed lemurs are medium-bodied, ecologically flexible primates that inhabit the southern reaches of Madagascar [4–6]. However, the ring-tailed lemur is under severe conservation pressure; it is classified as Endangered by the IUCN [7], resulting primarily from deforestation, hunting, and capture for the pet trade. A recent population census has revealed a dramatic population decline with as few as 2200 individuals remaining in the wild [8]. Of further concern, the species is distributed across a highly fragmented range with only eight populations of at least 100 individuals remaining [8]. Despite this near-term population decline, a recent microsatellite analysis indicates that the genetic diversity of *L. catta* populations could be exceptionally high, with evidence of genetic isolation by distance throughout their geographic range [6].

From a genomic perspective, relatively little is known about ring-tailed lemurs (and strepsirrhines more broadly). Genome assemblies have been published for 18 strepsirrhine species, but none of these assemblies has a contig N50 value above 1 Mb, and only three of them are above 100 kb [9]. Recently, a *Lemur catta* genome (LemCat_v1_BIUU) was assembled by the Zoonomia consortium [10], given that it is derived from illumina short reads, its metrics and application are still limited compared to the genome quality of recent highly contiguous assemblies [11]. This general lack of genomic resources remains a considerable limitation for the comparative and population genomics of lemurs.

Here, we present a new high-quality genome assembly of *L. catta* (mLemCat1) that conforms to the standards of the Vertebrate Genomes Project (VGP). mLemCat1 was assembled with a combination of PacBio continuous long reads (CLR reads), Optical Mapping Bionano reads, Arima HiC data, and 10X linked-reads. Our new assembly will allow for a deep assessment of the genome biology and conservation genomics of endangered ring-tailed lemurs. Additionally, given the paucity of high contiguity strepsirrhine assemblies, it will allow major advances in the genomics of across the Lemuridae family.

Data Description

Library preparation and sequencing

Spleen tissue was collected post-mortem from a male at the Copenhagen Zoo (Denmark) in 2015 and immediately flash-frozen (ZIMS Global Accession Number GAN: DKL15-03323). We isolated 30ug of ultra high molecular weight DNA (uHMW) from 35 mg of flash-frozen spleen tissue using the agarose plug Bionano Genomics protocol for animal tissue (DNA isolation fibrous tissue protocol (#30071C). uHMW DNA quality was assessed by a Pulsed Field Gel assay and quantified with a Qubit 2 Fluorometer.

10µg of uHMW DNA was sheared using a 26G blunt end needle (PacBio protocol PN 101-181-000 Version 05). A large-insert PacBio library was prepared using the Pacific Biosciences Express Template Prep Kit v2.0 (#100-938-900) following the manufacturer protocol. The library was then size selected (>20kb) using the Sage Science BluePippin Size-Selection System. 23 PacBio 1M v3 (#101-531-000) smrtcells were sequenced on the Sequel instrument (sequencing kit 3.0 #101-597-800) with a 10 hours movie and 2 hours pre-extension time. Unfragmented uHMW DNA was used to generate a linked-reads library on the 10X Genomics Chromium (Genome Library Kit & Gel Bead Kit v2 PN-120258, Genome Chip Kit v2 PN-120257, i7 Multiplex Kit PN-120262). This 10X library was sequenced on an Illumina Novaseq S4 150bp PE lane. uHMW DNA was labeled for Bionano Genomics optical mapping using the Bionano Prep Direct Label and Stain (DLS) Protocol (30206E) and run on one Saphyr instrument chip flowcell. Hi-C preparation was performed by Arima Genomics using the Arima-HiC kit (P/N: A510008) and an Illumina-compatible library was generated using the KAPA Hyper Prep kit (P/N: KK8504). This library was then sequenced on an Illumina HiSeq X (150bp PE) at ~60× coverage following the manufacturer's protocols. Assuming a genome size of 3.21 Gbp from the GoaT database [12], the present genome (mLemCat1) has 86.43X of 10.28X linked-reads data, 66.68X of Arima data, 154.57X of Bionano data and 62.88X of PacBio data.

De novo assembly

The genome was assembled following the VGP standard pipeline v1.6 [11]. Specifically, contigs were generated using FALCON [13] and FALCON-Unzip [14], producing a primary and an alternate assembly. We used purge_dups [15] to identify false duplications caused by regions of high-heterozygosity. Purged contigs were removed from the primary assembly and added to the alternate assembly. We then scaffolded the primary assembly using 10X linked-reads data with scaff10X 2.0 [16], Bionano optical maps with Bionano Solve v..2.1 [17], and Arima Hi-C data with Salsa 2.2 [18]. We assembled the mitochondrial genome separately using MitoVGP [19] with PacBio and 10X data. The primary scaffolds, alternate contigs, and mitochondrial assembly were polished simultaneously. We first performed Polishing and gap filling with the original PacBio data using Arrow [13], followed by two rounds of short-reads polishing using the 10X linked-reads data. Specifically, 10X data was mapped to the assembly using Longranger 2.1.3 [20] and polishing was done with FreeBayes [21]. All the computing was performed on the DNAnexus cloud platform (https://www.dnanexus.com).

Genome Quality Assessment

Compared to the currently available short-read *Lemur catta* genome available (LemCat_v1_BIUU) [10], the new mLemCat1 assembly has higher contiguity values, fewer scaffolds, and a slightly smaller assembly size (Table 1). We generated basic continuity assembly metrics for both assemblies using QUAST V5.0.2 (QUAST, RRID:SCR_001228) [22], which are presented in Table 1. The assembly has a total scaffold size of 2.122 Gb within 141 scaffolds. The mLemCat1 contig and scaffold N50 values are 10.570 Mb, and 90.982 Mb, representing 20.41 fold and 421.21 fold increases, respectively, compared to the LemCat_v1_BIUU assembly. The overall GC content of this assembly is 40.48%.

The mLemCat1 assembly has a high level of accuracy and completeness that conforms to the proposed standards of the VGP [11]. We assessed the base and structural accuracies of the assembly with Merqury V1.1, using a Meryl V1.7 database [23] based on 130.708 Gb (84X coverage) of 10x linked-reads reads. The base pair QV of the primary assembly is 44.35, which exceeds the VGP standard. The k-mer completeness is 91.45%. We classified the structural accuracy using the false duplications percentage calculated in the *false_duplications.sh* script from Merqury V1.1. The assembly is estimated to have 0.39% false duplications based on the percentage of kmers found in unexpected copy numbers.

Table 1: Genome Quality Metrics for the mLemCat1 genome assembly compared to previous assembly and standards. S: single-copy genes, MT: mitochondrial; Gbp: giga base pairs; Mbp: mega base pairs; #: number.

QUALITY CATEGORY	QUALITY METRIC	VGP STANDARD	mLemCat1	LemCat_v1_BIUU
**Continuity**	# Scaffolds	-	141	575,427
	Scaffold N50	23-480 Mbp	90.982 Mbp	0.216 Mbp
	Largest scaffold	-	285.823 Mbp	2.320 Mbp
	# Contigs	-	518	580,026
	Contig N50	1-25 Mbp	10.570 Mbp	0.158 Mbp
	Largest contig	-	40.360 Mbp	1.312 Mbp

	Gaps / Gbp	75-1500	≈ 179.5	≈ 2001.3
	Span	-	2.122 Gbp	2.298 Gbp
**Structural accuracy**	False duplications	0.2-5.0%	0.39%	-
**Base accuracy**	Base pair QV	39-43	44.45	-
	K-mer completeness	87-98%	91.45%	-
**Functional completeness**	Genes (BUSCOs (S))	82-98%	88.80%	81.46%
**Chromosome status**	Organelles (e.g. MT)	1 Complete allele	1 Complete allele	-

In order to assess the functional completeness of the assembly, we recovered BUSCO genes from both mLemCat1 and the existing Illumina-based assembly (LemCat_v1_BIUU) (Figure 2). Specifically, we conducted a gene completeness assessment using BUSCO V4.0.6 [24], setting human as the reference species in the --augustus_species parameter, and using the primates_OrthoDB10 database (https://busco.ezlab.org/), which comprises a total of 13780 genes. Of the 13780 possible BUSCOs, we identified 12138 single-copy (88.8%), 100 duplicates (0.7%), and 188 fragmented genes (1.4%) in mLemCat1, leaving 9.8% of BUSCOs missing. In contrast, we could only recover 11132 single-copy BUSCOs (81.5%) from LemCat_v1_BIUU, with 15.3% of BUSCO genes missing.

Mitogenome of L. catta

We assembled a gapless mitochondrial genome with a span of 17086 bp, and annotated the assembly using the *MITOS2 web server* [25]. With the annotation results we plotted a map of the mitochondrion with GenomeVx [26] (Figure S1). Thirteen main protein coding genes have been annotated in this new mitogenome including nad1, nad2, nad3, nad4, nad4L, nad5, nad6, cox1, cox2, cox3, atp6, atp8 and cob.

Analysis of the repeatome

To assess the structure and variety of repeat elements in the *L. catta* genome, we analyzed mLemCat1 with RepeatMasker 4.1.2-p1. Non-default settings included the use of sensitive mode, the query assumed species set to primates, nhmmscan 3.3.2 (Nov 2020), and FamDB: HMM-Dfam_3.3, without the exclusion of simple repeats. In total, 50.32% of the bases in the *L.*

catta genome (mLemCat1) are masked as interspersed repeats, including LINEs, SINEs, LTRs and DNA elements (Figure 3A, Table S1). In general terms, the portion of the genome that comprises repetitive elements is similar to that which has been reported for other high-quality catarrhine genomes [27,28], although there are fewer satellites (0.30%), simple repeats (0.68%) and low complexity elements (0.13) (Table S1).

In comparison with the previous illumina-only assembly (LemCat_v1_BIUU) we observed minor differences in the structure and variety of repeat elements (Figure 4). The new long-read based assembly has 1.31% more interspersed repeats (50.32% vs 49.01%), and a higher percentage of sequence in each repeat subtype, except for satellites, simple repeats, low complexity elements, and ERV classes I & II. We also observed both a lower percentage of sequence and a smaller number of ALU events in mLemCat1. Additionally, the total number of masked bases is lower in the new assembly, but they represent a higher percentage of the sequence, due to mLemCat1 having a shorter span.

Alus are the most abundant repeat elements in the human genome, and differences in their rates, distribution, and proliferation could have led to distinct functional changes in multiple primate lineages [30]. Alu elements have been present since the earliest stages of primate evolution are frequently located in gene-rich regions, and may have an important role in gene regulation [30–33]. In order to compare the Alu repeat landscape of *L. catta* with those of other highly-contiguous primate assemblies, we ran RepeatMasker as above adding the *-alu* option. The genomes used for the comparison were long-read based assemblies, including human (hg38), chimpanzee (panTro6), western gorilla (gorGor6), Sumatran orangutan (ponAbe3), rhesus macaque (rheMac10), common marmoset (calJac4), and gray mouse lemur (Mmur_3.0) (table S2).

We identified substantially fewer Alu elements in the lemur genomes (*L. catta* and *Microcebus murinus*) than those of the catarrhines, with the fewest being found in the *L. catta* genome (3.66% of repeat elements) (figure 3B, table S3). In contrast to the other primates assessed, for which AluS elements are most abundant, AluJ is the most common element in mLemCat1 (54.17% of Alu events). Both lemurs have fewer AluS events than the anthropoids and fewer AluY events than the catarrhines, consistent with previous reports of the expansion of these two families after the Catarrhini-Strepsirrhini split [34]. The fact that the common marmoset has the highest number of AluS elements (figure 3C) confirms that the burst that started before the Catarrhini and Platyrrhini parvorders diverged, continued with different activity in both lineages after their split. Recent Alu activity (AluY events), is most abundant in catarrhines, particularly the rhesus macaque, which when compared to great apes (figure 3C), has a higher overall percentage of Alus (figure 3B).

CONCLUSION

We have assembled a new high-quality genome reference for the ring-tailed lemur (*L. catta*) that satisfies the VGP quality assembly standards. Compared to pre-existing genomic resources, the new assembly has higher contiguity and completeness, and contains more single copy complete BUSCO genes with fewer fragmented or missing genes. Additionally, we analyzed the *L. catta* repeatome and observed substantially fewer Alu events compared to other high-quality primate assemblies. This assembly illustrates how long-reads and further scaffolding data such as HiC or optical mappings can drastically improve the contiguity and completeness of an assembly, which also allows for improved analysis of structural variation. We suggest that this new assembly will be an excellent resource for the mammalian genomics community, with particular value for the conservation genomics of lemurs.

DATA AVAILABILITY

mLemCat1 assembly and the raw reads used to generate it can be accessed at GenomeArk https://vgp.github.io/genomeark/Lemur_catta/. Accessed 04 Oct 2021. The complete mitogenome of mLemCat1 is available in Genomeark as mLemCat1.MT.20190820.fasta.gz https://vgp.github.io/genomeark/Lemur_catta/. Accessed 04 Oct 2021.

COMPETING INTERESTS

The authors declare that they have no competing interests.

AUTHOR CONTRIBUTIONS

MPF and JDO analyzed the data; JM and BH generated the data; BH generated the draft assembly; MFB collected the samples. MPF and JDO wrote the paper with contributions from all authors. TMB, EFJ, and OF designed the research.

ACKNOWLEDGEMENTS

The project that gave rise to these results received the support of two fellowship from ”la Caixa” Foundation (ID 100010434) and from the European Union’s Horizon 2020 research and innovation programme under the Marie Skłodowska-Curie grant agreement No 847648. The fellowship codes are LCF/BQ/PI20/11760004 & LCF/BQ/DR20/11790032. TMB is supported by funding from the European Research Council (ERC) under the European Union’s Horizon 2020 research and innovation programme (grant agreement No. 864203), BFU2017-86471-P (MINECO/FEDER, UE), “Unidad de Excelencia María de Maeztu”, funded by the AEI (CEX2018-000792-M), Howard Hughes International Early Career, NIH 1R01HG010898-01A1 and Secretaria d’Universitats i Recerca and CERCA Programme del Departament d’Economia i Coneixement de la Generalitat de Catalunya (GRC 2017 SGR 880).

REFERENCES

1. Estrada A, Garber PA, Rylands AB, Roos C, Fernandez-Duque E, Di Fiore A, et al.. Impending extinction crisis of the world's primates: Why primates matter. *Sci Adv*. 3:e16009462017;

2. Orkin JD, Kuderna LFK, Marques-Bonet T. The Diversity of Primates: From Biomedicine to Conservation Genomics. *Annu Rev Anim Biosci*. 9:103–242021;

3. Fleagle JG. Primate Adaptation and Evolution. Academic Press;

4. Sussman RW. Demography and social organization of free-rangingLemur catta in the Beza Mahafaly Reserve, Madagascar. *Am J Phys Anthropol*. Wiley; 84:43–581991;

5. Cameron A, Gould L. Fragment-Adaptive Behavioural Strategies and Intersite Variation in the Ring-Tailed Lemur (Lemur catta) in South-Central Madagascar. In: Marsh LK, Chapman CA, editors. *Primates in Fragments: Complexity and Resilience*. New York, NY: Springer New York; p. 227–43.

6. Chandrashekar A, Knierim JA, Khan S, Raboin DL, Venkatesh S, Clarke TA, et al.. Genetic population structure of endangered ring-tailed lemurs (Lemur catta) from nine sites in southern Madagascar. *Ecol Evol*. 10:8030–432020;

7. Lafleur M, Gould L. Lemur catta. *The IUCN Red List of Threatened Species*. 2020; doi: 10.2305/IUCN.UK.2020-2.RLTS.T11496A115565760.en.

8. LaFleur M, Clarke TA, Reuter K, Schaeffer T. Rapid Decrease in Populations of Wild Ring-Tailed Lemurs (***Lemur catta***) in Madagascar. *Folia Primatol* . 87:320–302016;

9. Kuderna LF, Esteller-Cucala P, Marques-Bonet T. Branching out: what omics can tell us about primate evolution. *Curr Opin Genet Dev*. 62:65–712020;

10. Zoonomia Consortium. A comparative genomics multitool for scientific discovery and conservation. *Nature*. 587:240–52020;

11. Rhie A, McCarthy SA, Fedrigo O, Damas J, Formenti G, Koren S, et al.. Towards complete and error-free genome assemblies of all vertebrate species. *Nature*. Nature Publishing Group; 592:737–462021;

12. Challis RJ, Kumar S, Stevens L, Blaxter M. GenomeHubs: simple containerized setup of a custom Ensembl database and web server for any species. *Database* . 2017; doi: 10.1093/database/bax039.

13. Chin C-S, Alexander DH, Marks P, Klammer AA, Drake J, Heiner C, et al.. Nonhybrid, finished microbial genome assemblies from long-read SMRT sequencing data. *Nat Methods*. 10:563–92013;

14. Chin C-S, Peluso P, Sedlazeck FJ, Nattestad M, Concepcion GT, Clum A, et al.. Phased diploid genome assembly with single-molecule real-time sequencing. *Nat Methods*. 13:1050–42016;

15. Guan D, McCarthy SA, Wood J, Howe K, Wang Y, Durbin R. Identifying and removing haplotypic duplication in primary genome assemblies. *Bioinformatics*. 36:2896–82020;

16. Ning Z, Harry E: Scaff10X. https://github.com/wtsi-hpag/Scaff10X Accessed 2021.

17. Bionano Genomics, Inc: Bionano Software Downloads. https://bionanogenomics.com/support/software-downloads/ Accessed 2021.

18. Ghurye J, Rhie A, Walenz BP, Schmitt A, Selvaraj S, Pop M, et al.. Integrating Hi-C links with assembly graphs for chromosome-scale assembly.

19. Formenti G, Rhie A, Balacco J, Haase B, Mountcastle J, Fedrigo O, et al.. Complete vertebrate mitogenomes reveal widespread repeats and gene duplications. *Genome Biol*. 22:1202021;

20. Bishara A, Liu Y, Weng Z, Kashef-Haghighi D, Newburger DE, West R, et al.. Read clouds uncover variation in complex regions of the human genome. Genome Research.

21. Garrison E, Marth G. Haplotype-based variant detection from short-read sequencing. arXiv [q-bio.GN].

22. Gurevich A, Saveliev V, Vyahhi N, Tesler G. QUAST: quality assessment tool for genome assemblies. *Bioinformatics*. 29:1072–52013;

23. Rhie A, Walenz BP, Koren S, Phillippy AM. Merqury: reference-free quality, completeness, and phasing assessment for genome assemblies. *Genome Biol*. 21:2452020;

24. Simão FA, Waterhouse RM, Ioannidis P, Kriventseva EV, Zdobnov EM. BUSCO: assessing genome assembly and annotation completeness with single-copy orthologs. *Bioinformatics*. 31:3210–22015;

25. Donath A, Jühling F, Al-Arab M, Bernhart SH, Reinhardt F, Stadler PF, et al.. Improved annotation of protein-coding genes boundaries in metazoan mitochondrial genomes. *Nucleic Acids Res*. Oxford Academic; 47:10543–522019;

26. Conant GC, Wolfe KH. GenomeVx: simple web-based creation of editable circular chromosome maps. *Bioinformatics*. academic.oup.com; 2008;

27. He Y, Luo X, Zhou B, Hu T, Meng X, Audano PA, et al.. Long-read assembly of the Chinese rhesus macaque genome and identification of ape-specific structural variants. *Nat Commun*. 10:1–142019;

28. Kronenberg ZN, Fiddes IT, Gordon D, Murali S, Cantsilieris S, Meyerson OS, et al.. High-resolution comparative analysis of great ape genomes. *Science*. 2018; doi: 10.1126/science.aar6343.

29. Batzer MA, Deininger PL, Hellmann-Blumberg U, Jurka J, Labuda D, Rubin CM, et al.. Standardized nomenclature for Alu repeats. *J Mol Evol*. 42:3–61996;

30. Liu GE, Alkan C, Jiang L, Zhao S, Eichler EE. Comparative analysis of Alu repeats in primate genomes. *Genome Res*. genome.cshlp.org; 2009;

31. Urrutia AO, Ocaña LB, Hurst LD. Do Alu repeats drive the evolution of the primate transcriptome? *Genome Biol*. 9:R252008;

32. Oliver KR, Greene WK. Mobile DNA and the TE-Thrust hypothesis: supporting evidence from the primates. *Mob DNA*. 2:82011;

33. Kurosaki T, Ueda S, Ishida T, Abe K, Ohno K, Matsuura T. The Unstable CCTG Repeat Responsible for Myotonic Dystrophy Type 2 Originates from an AluSx Element Insertion into an Early Primate Genome. PLoS ONE.

34. Konkel MK, Walker JA, Batzer MA. LINEs and SINEs of primate evolution. *Evol Anthropol*. 19:236–492010;

FIGURE LEGENDS

Figure 1: Ring-tailed lemur (*L. catta*); photo courtesy of Copenhagen Zoo

Figure 2: BUSCO Assessment Results comparison between mLemCat1 and LemCat_v1_BIUU *Lemur catta* assemblies using the Primates_ODB10 database (n = 13780). The new mLemCat1 assembly shows a 7.3% increase in complete single copy orthologous genes.

Figure 3: A) Percentages of elements in the *L. catta* genome (mLemCat1) masked by RepeatMasker. B) Percentage of Alus masked in primate long-read assemblies. C) Spider plots of the total number of different Alu-like elements masked in each genome assembly. Lemurs have fewer AluS elements than anthropoid primates. Axis values represent 1,000x events. FAM (Fossil Alu Monomer); FLAMs (Free Left Alu Monomers); FRAMs (Free Right Alu Monomers); AluJ (oldest); AluS (intermediate); AluY (youngest); Alu (non-specified) [29]

Figure 4: Comparison of repeat variety and structure between mLemCat1 and LemCat_V1_BIUU assemblies.

Figure3

Click here to access/download;Figure;Figure3_Repeats.pdf

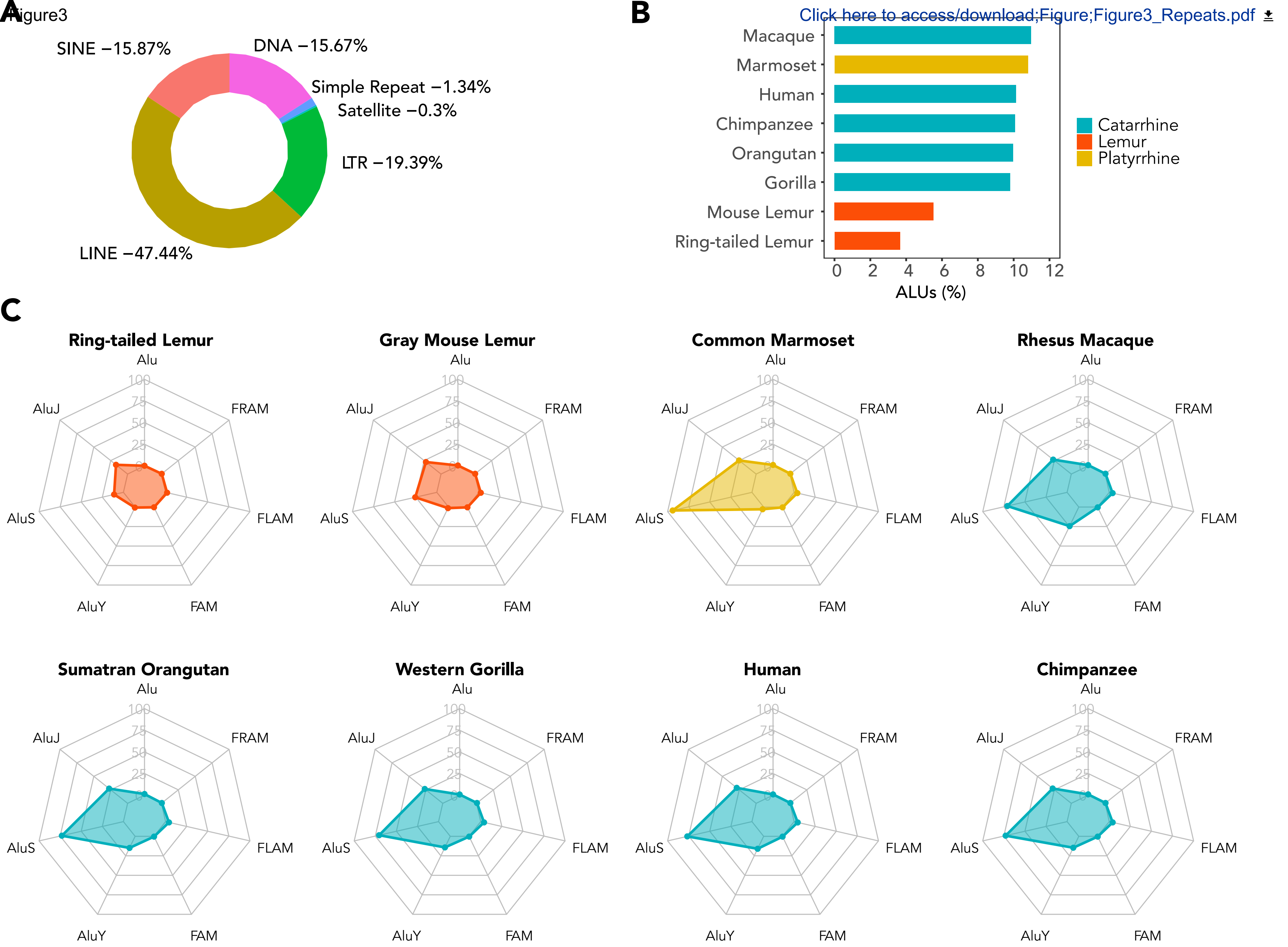

Figure4

Click here to access/download;Figure;Figure4_RepeatComparisonPlot.pd

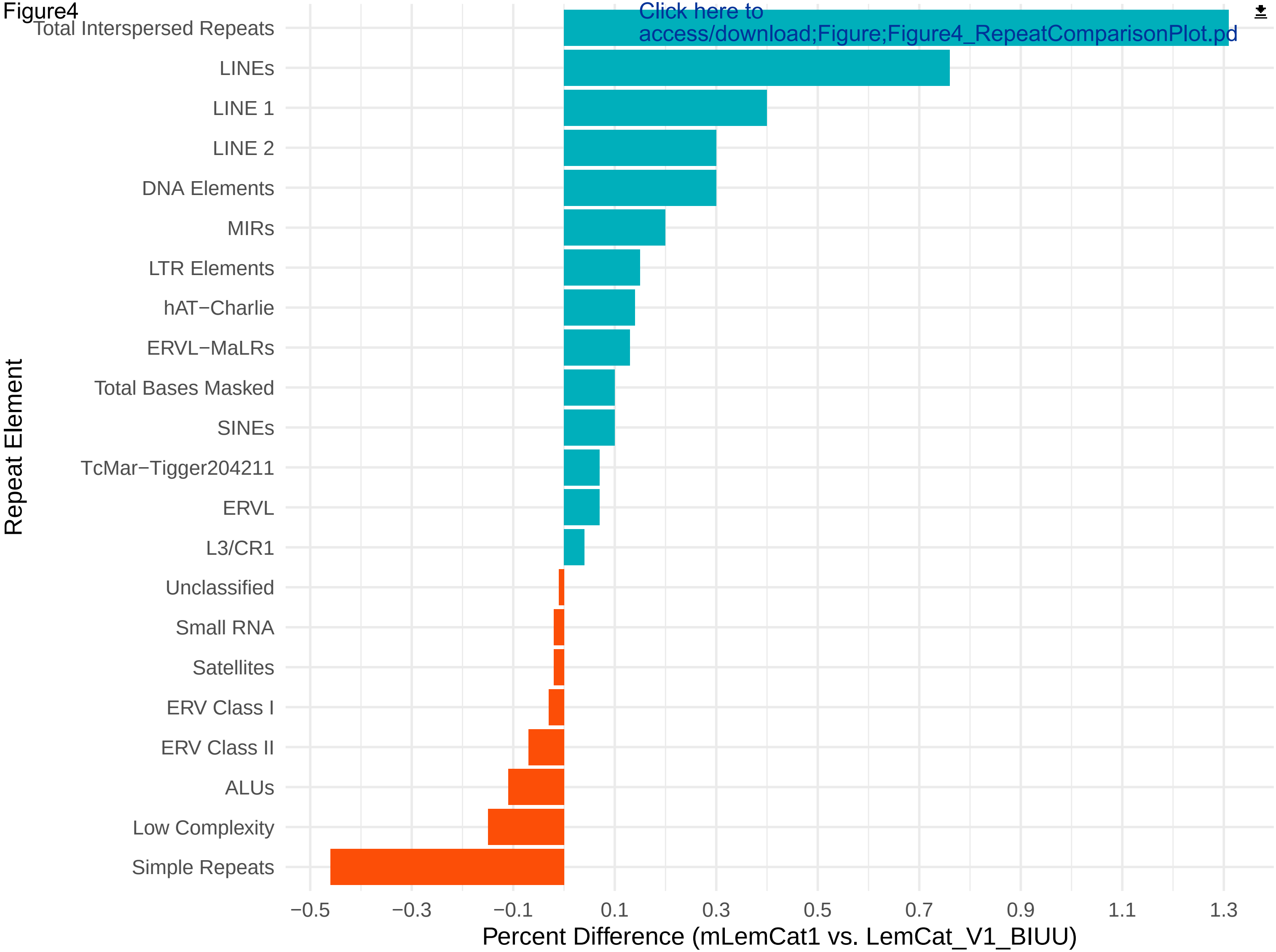

Figure2

Click here to access/download;Figure;Figure2_BUSCOs.pdf

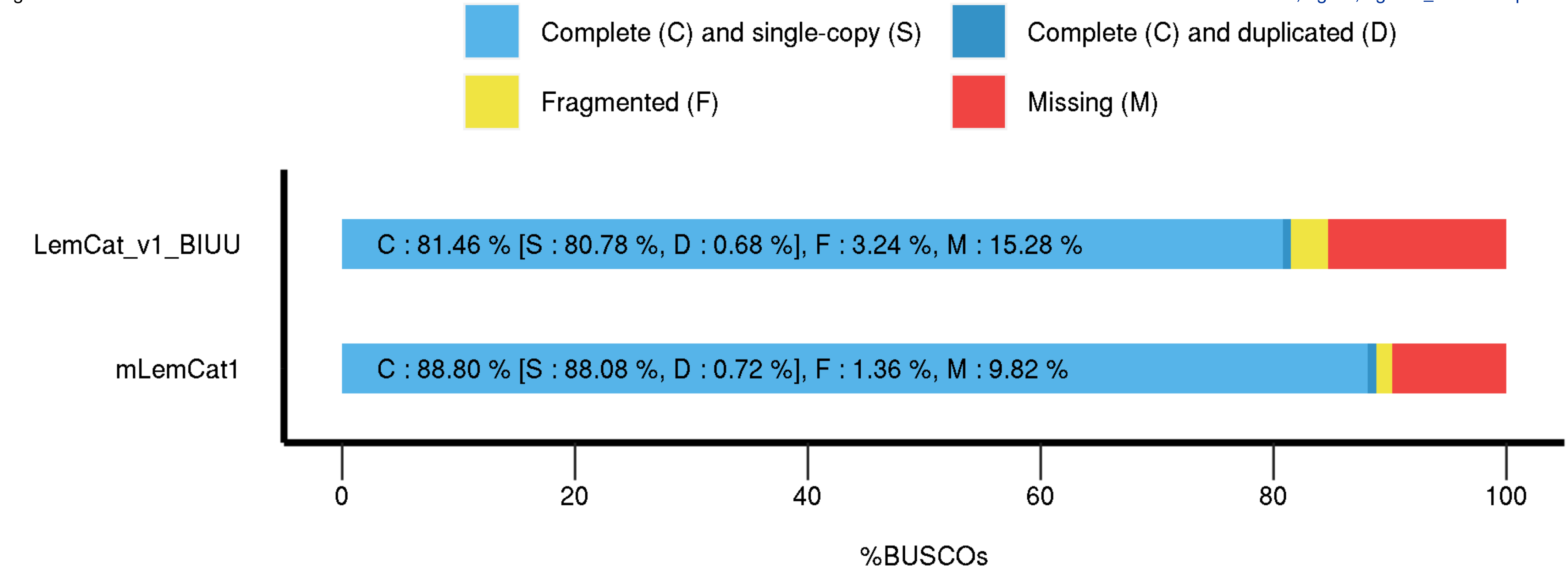

Figure1

Click here to access/download;Figure;Figure1_Lemurcatta.pdf

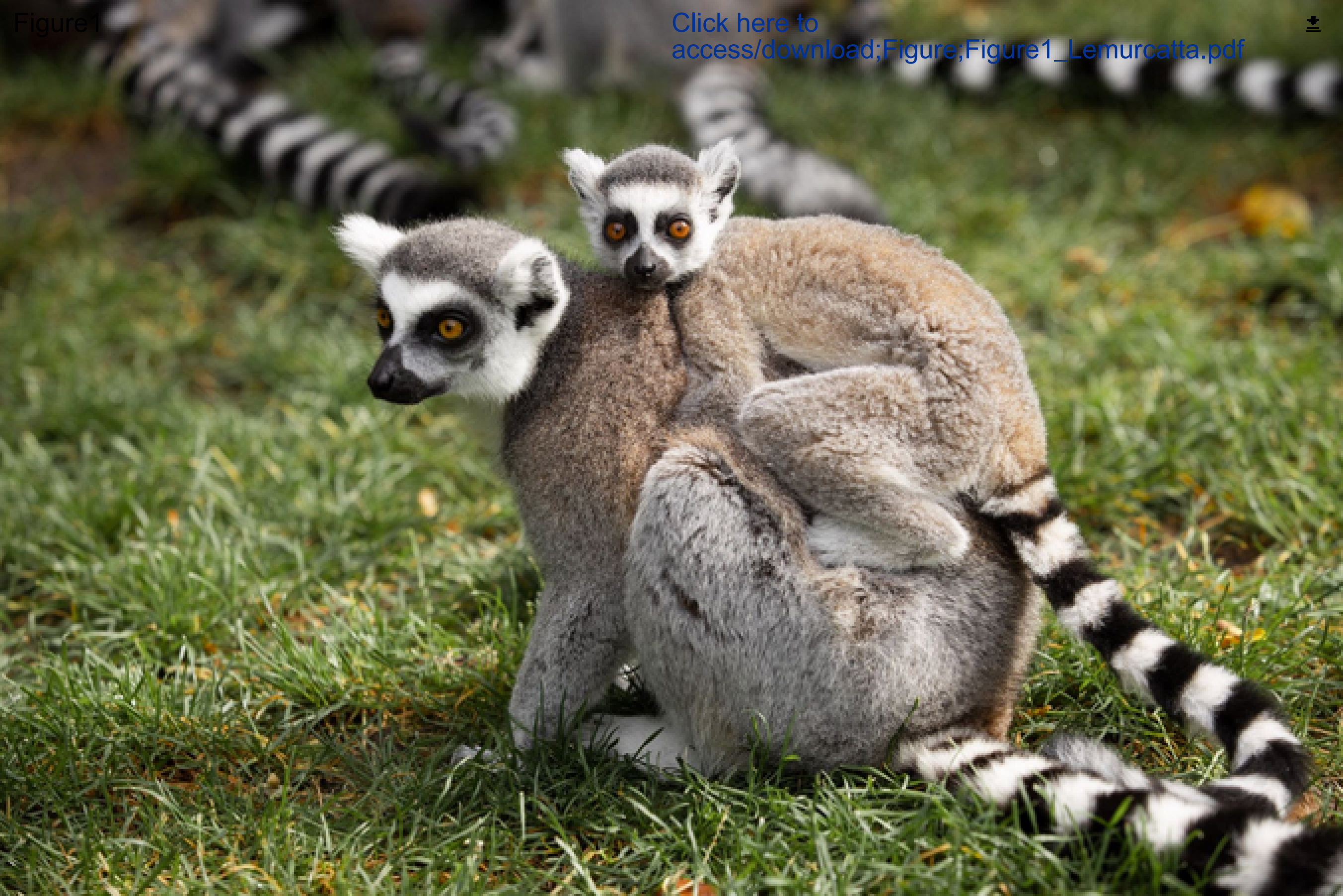

Click here to access/download
Supplementary Material
LemurCattaRef_Supplemental.docx
