# Supplementary material for: A high-quality, long-read genome assembly of the endangered ring-tailed lemur (Lemur catta)
Source: Gigascience. 2022 Apr 1;11:giac026. doi: 10.1093/gigascience/giac026 (PMC8975718; doi:10.1093/gigascience/giac026)
Supplement: giac026_GIGA-D-21-00335_Revision_1 [file giac026_giga-d-21-00335_revision_1.pdf]

GigaScience

Data Note: A high-quality, long-read genome assembly of the endangered ring-tailed lemur (Lemur catta)

--Manuscript Draft--

**Manuscript Number:**	GIGA-D-21-00335R1
**Full Title:**	Data Note: A high-quality, long-read genome assembly of the endangered ring-tailed lemur (Lemur catta)
**Article Type:**	Data Note
**Funding Information:**	"la Caixa" Foundation (LCF/BQ/PI20/11760004) — Dr. Joseph D. Orkin
	"la Caixa" Foundation (LCF/BQ/DR20/11790032) — MSc Marc Palmada-Flores
	H2020 European Research Council (864203) — Dr. Tomas Marques-Bonet
	H2020 Marie Skłodowska-Curie Actions (847648) — Dr. Joseph D. Orkin
	"Unidad de Excelencia María de Maeztu" (CEX2018-000792-M) — Dr. Tomas Marques-Bonet
	Howard Hughes Medical Institute (NIH 1R01HG010898-01A1) — Dr. Tomas Marques-Bonet
	Departament d'Economia i Coneixement de la Generalitat de Catalunya (GRC 2017 SGR 880) — Dr. Tomas Marques-Bonet
**Abstract:**	The ring-tailed lemur (Lemur catta) is a charismatic strepsirrhine primate endemic to the island of Madagascar. These lemurs are of particular interest, given their status as a flagship species and widespread publicility in popular media. Unfortunately, a recent population decline has caused the census population to fall below 2500 individuals in the wild, and their classification as an endangered species by the IUCN. As is the case for most strepsirrhine primates, only a limited amount of genomic research has been conducted on L. catta , in part due to the lack of genomic resources. We generated a new high-quality reference genome assembly for L. catta (mLemCat1) that conforms to the standards of the Vertebrate Genomes Project. This new long-read assembly is composed of PacBio continuous long reads (CLR reads), Optical Mapping Bionano reads, Arima HiC data, and 10X linked-reads. The contiguity and completeness of the assembly is extremely high, with scaffold and contig N50 values of 90.982 Mbp and 10.570 Mbp, respectively. Additionally, when compared to other high-quality primate assemblies, L. catta has the lowest reported number of Alu elements, which results predominantly from a lack of AluS and AluY elements. mLemCat1 is an excellent genomic resource not only for the ring-tailed lemur community, but also for other members of the Lemuridae family and is the first very long read assembly for a strepsirrhine.
**Corresponding Author:**	Marc Palmada-Flores<br>Institute of Evolutionary Biology: Instituto de Biologia Evolutiva<br>Barcelona, SPAIN
**Corresponding Author Secondary Information:**	
**Corresponding Author's Institution:**	Institute of Evolutionary Biology: Instituto de Biologia Evolutiva
**Corresponding Author's Secondary Institution:**	
**First Author:**	Marc Palmada-Flores
**First Author Secondary Information:**	
**Order of Authors:**	Marc Palmada-Flores
	Joseph D. Orkin
	Bettina Haase

Powered by Editorial Manager® and ProduXion Manager® from Aries Systems Corporation

	Jacquelyn Mountcastle
	Mads F. Bertelsen
	Olivier Fedrigo
	Lukas Kuderna
	Erich D. Jarvis
	Tomas Marques-Bonet
**Order of Authors Secondary Information:**	
**Response to Reviewers:**	GIGA-D-21-00335<br>Data Note: A high-quality, long-read genome assembly of the endangered ring-tailed lemur (Lemur catta)<br>Marc Palmada-Flores; Joseph D. Orkin; Bettina Haase; Jacquelyn Mountcastle; Mads F. Bertelsen; Olivier Fedrigo; Lukas Kuderna; Erich D. Jarvis; Tomas Marques-Bonet<br>GigaScience<br><br>**************************<br><br>Response to Editor:<br><br>Your manuscript "Data Note: A high-quality, long-read genome assembly of the endangered ring-tailed lemur (Lemur catta)" (GIGA-D-21-00335) has been assessed by our reviewers. Based on these reports, and my own assessment as Editor, I am pleased to inform you that it is potentially acceptable for publication in GigaScience, once you have carried out some essential revisions suggested by our reviewers.<br><br>We thank the Editor for inviting a resubmission and for the supportive words about our study and our approach. Below, we detail how we have responded to each of the constructive points raised by the reviewers. We believe our manuscript is improved through addressing these numerous helpful points and we now hope you find it suitable for publication in GigaScience.<br><br>**************************<br><br>Response to Reviewers:<br><br>Reviewer #1: The manuscript of "A high-quality, long-read genome assembly of the endangered ring-tailed lemur (Lemur catta)" reports a updated genome assembly for ring-tailed lemur (Lemur catta), a Strepsirrhine primate species. In combination with PacBio continuous long reads (CLR reads), Bionano reads, HiC data, and 10X linked-reads, the contig and scaffold N50 in the newly acquired genome assembly each reached to 10.570 Mbp and 90.982 Mbp. This genome assembly statistic represents 20.41 fold and 421.21 fold increases, respectively, which high quality reference genome could be served as a valuable data resource compared with the previous short-read genome of the species. As the first reported long read assembly for a Lemuriformes, one infraorder within Strepsirrhine, this genomic resource distinguished with previous report which typically focused on higher-primate, especially the apes and old-world monkeys. The release of this genome could potentially facilitate further comparable genomic analysis, help on the understanding of adaptive evolution in primates from Strepsirrhine to Haplorrhini. This updated genome is expected to gain more attention in the research areas of comparative genomics, genetics, conservation and behavior in primates as well as mammals.<br>The manuscript is well written, technically correct. I suggest accept this paper after minor revision.<br><br>Some questions belowing may be helpful to improve the manuscript.<br><br>We are very grateful to the reviewer for the positive assessment of our manuscript and welcome the suggestion of acceptance after minor revisions. Please take note of our responses to the specific questions below.

Powered by Editorial Manager® and ProduXion Manager® from Aries Systems Corporation

	1. In the introduction section, beside background of distribution and taxonomy of ring-tailed lemurs, more information will be appreciate including phylogeny position and their biological background such as diet, behavior on so on.<br><br>Thank you for this suggestion. We have extended the introductory paragraph to include the following text about ring-tailed lemur ecology and phylogenetic positioning.<br><br>"Ring-tailed lemurs are medium-bodied, ecologically flexible members of the Lemuridae family and the sole member of the genus Lemur. In contrast to most other Lemuridae, L. catta predominantly inhabit the dry and seasonal forests of southern Madagascar [1]. They consume an omnivorous diet mostly of fruit and leaves, and engage in a multi-male multi-female social structure with a polygynandrous mating system [1]."<br><br>2. During the de novo assembly and subsequent analysis, the authors use several different software packages for their analysis. However, the specific parameter settings for the software used were not given.<br><br>Thank you for drawing our attention to this issue, which we have now clarified in the text and added in Additional File 1. In order to keep the text concise, we had not listed every parameter and setting explicitly in the text. However, we have now included a link to the VGP master pipeline in the "De novo assembly" section, which provides these details. All the parameters used for the assembly pipeline can be found in the VGP github, from which our pipeline is derived and includes all the scripts and parameters used. The following websites will be added in the Additional File 1.<br><br>https://github.com/VGP/vgp-assembly/tree/master/pipeline<br>For example, for the bionano scaffolding step the config.xml file: https://github.com/VGP/vgp-assembly/blob/master/pipeline/bionano/hybridScaffold_DLE1_config.xml<br>For salsa we used the default parameters: https://github.com/VGP/vgp-assembly/blob/master/pipeline/salsa/salsa2.2.sh<br>For 10X scaffolding, you can see the parameters used here: https://github.com/VGP/vgp-assembly/blob/master/pipeline/scaff10x/scaff10x.sh<br>The falcon unzip parameters can be found here: https://github.com/VGP/vgp-assembly/blob/master/dx_workflows/vgp_falcon_and_unzip_assembly_workflow/dxworkflow.json<br><br>The assembly pipeline was run on DNanexus, with the default parameters and the reads filtered using "min_read_length": 500 and "target_coverage": 50.<br><br>The remaining software specific parameters are now present in the text. All RepeatMasker analyses are embedded in the text and commands have been added to Additional File 1.<br><br>BUSCOs parameters are also specified in the text and commands have been added to Additional File 1.<br><br>The MITOS2 server ran the annotation of the mitogenome with the default parameters.<br><br>3. The detailed scaffolding step was also missed for the Arima Hi-C data with Salsa 2.2 [18]. How authors deal with the sequence order? This information could help us to understand how the authors addressed the technical issue such as orientation for the inversion regions within the scaffolds.<br><br>Thanks for pointing out this matter. The sequence order is not something we considered specifically, but we suggest that these technical issues should not cause any substantial problems for our assembly, given that the contigs we assembled are of exceptionally long lengths and we used two types of scaffolding technology data, with which the types of errors proposed by the reviewer are unlikely to affect our assembly. Specifically, SALSA2 software paper [2] explains how short contigs lead to higher amounts of misoriented contigs within scaffolds, and outperforms its previous version in this regard.

Powered by Editorial Manager® and ProduXion Manager® from Aries Systems Corporation

4. The gapless mitochondrial genomes were assembled by PacBio long reads and 10X short reads, and were annotated the by using the MITOS2 web server. The short sequencing reads were typically chosen and used for most mitochondrial genome assembly. Please explain why both the long reads and short reads were chosen during the assembly, or whether this combined strategy presents any advantages compare with traditional method? In addition, in the annotation process for mitogenome, MITOS2 web server was employed, but the descriptions of the procedures could not been found. The details how to reorder and concatenate the annotated genes and regions are appriciate.

The reviewer raises an important point, and we should have been more clear about it in the manuscript. Details regarding the mitogenome assembly process were recently published (Formenti et al. 2021) as part of the broader mitoVGP pipeline, which we have now clarified and cited. The advantage of our combined short-and-long read strategy is that the highly repetitive nature of the mitochondrial control region (CR) sometimes does not allow for complete error-free assemblies of the mitogenome using short-read data alone. In this specific case there is a small repeat region which is correctly assembled using both long and short reads to obtain the complete mitogenome. We have added the corresponding explanation and citation in the main text.

For the annotation we used MITOS2, a web server that easily annotates genes and regions of any mitochondrial genome. Further details on the procedures can be found in [3], and the corresponding github repository (https://github.com/gavieira/mitos2_wrapper), where you will find the code and specifics of the software, which we did not modify.

5. Please format the references into same style. For example, in reference 19, vs. reference 20. Please revise all "Lemur catta" into italic. Please check and revise according to the policy of GigaScience.

We apologize for this oversight. All references have now been correctly formatted according to GigaScience policy using reference software.

6. Did the author confused the order between Figure 3 and Figure 4?

We apologize for the confusion. We have now reordered the figures during the submission process of the manuscript.

Reviewer #2:

This is a great work conducting genome assembly of this primate species. The assembly would highly benefit from the annotation of the genome (gene annotation) using RNA seq data, however, this seems to be beyond the goals of this manuscript. Since the focus of the study is on the genome assembly, it would be helpful to conduct Chrimosome Synteny analysis with human genome and other primate species to give a big picture of the differences across the species.
Below, please the comments to this work.

We very much appreciate the reviewer’s kind response and positive assessment. The suggestion of a chromosome synteny analysis is an excellent one, which is described below.

Abstract:

Continuous Long Read (CLR) NOT (CLR Reads)? Isn't the word "Read" already included in the abbreviation? Not sure what is the standard abbreviation for this term, and if it really needs mentioning the word "Read".

Thank you for pointing out this oversight. We have adjusted both references from “(CLR reads)” to “(CLR data)”. “CLR reads” is a commonly used expression in the field, but we agreed that changing it to “CLR data” makes more sense.

Powered by Editorial Manager® and ProduXion Manager® from Aries Systems Corporation

Data Description:

* Any data on the quality of HMW DNA evaluation? Would be good to cite this data in the first paragraph of the Data Description where the authors mention HMW DNA quality control.

We used a PFGE gel (Sage Pippin Pulse) as a HMW quality control measure. We have added the corresponding image as a supplementary figure ("Figure S1: Pulse Field Gel assay (Sage Pippin Pulse) with HMW ladder used for quality control of the ultra-High Molecular Weight DNA (Lemur catta is in well number 1)") and the corresponding text (“uHMW DNA quality was assessed by a Pulsed Field Gel assay and quantified with a Qubit 2 Fluorometer (Figure S1)”) in the manuscript as suggested.

* Would be great for the authors to report the results of repeat analysis using Repeat Modeler.

Thank you for this suggestion, which we have given substantial consideration. We decided to run RepeatMasker exclusively for several reasons, but primarily, because there is a high likelihood that a comparable outcome would be produced by RepeatModeler. Additionally, RepeatModeler’s results would lack power for comparison between species, because it depends directly on the quality of the assemblies used. More specifically, running RepeatModeler requires the use of a previously established repeat library in order to classify the repeats present in the focal genome to obtain a specific database of repeats for the genome masking. The standard library in this case would be Dfam, which is also what we used for our RepeatMasker run to classify repeats. We suggest that the well-established primates database provided by RepeatMasker, which is derived from a larger number of genomes, is an appropriate choice for the masking of a lemur genome; thus, we are more confident in our results than we otherwise would be by creating a new database based solely on the present genome. Secondly, RepeatModeler is a well-known and commonly used software and the already complete database it provides will allow for more systematic comparison and analysis by other researchers. Creating a database based on the Lemur catta genome alone could help to find specific repeat patterns within the species, but ultimately, it would still be based on the same previously known library of repeats that RepeatMasker uses to classify them. As such, we think that the computational hurdle of running RepeatModeler would not substantially alter our results.

* Any Synteny analysis compared to other primate species? One of the most useful information from a long-read sequencing (and chromosome-level assembly) is the ability to compare the chromosomal synteny with other primates (or just with humans).

We thank the reviewer for drawing our attention to this issue, and agree that this assembly can be a powerful tool for chromosomal comparison and finding syntenies between Lemur catta and other species. For this purpose, we did a synteny analysis creating a dot plot using Mummer v3.23 software’s nucmer -mum option and visualized the results of the synteny between the present assembly of Lemur catta (mLemCat1) and an assembly of Homo sapiens (hg38) using the https://dot.sandbox.bio/ website. We have added this synteny plot as a supplementary figure and the following text to the manuscript:

“The present assembly (mLemCat1) can be useful to create synteny plots between L. catta and others, such as humans (Figure S2), as it has N50 statistics comparable to other high-quality primate genomes recently published (Table S2).”

We added the Figure S2: An overall chromosomal synteny plot between Lemur catta (mLemCat1 assembly) and Homo sapiens (hg38 assembly) in the supplementary material file.

* What is the number of scaffolds that cover 90% of the genome? How different is this number (the number of scaffolds that cover 90% of the genome) compared to the number of chromosomes for this species? Also, what about N95? Would be good to discuss these statistics more clearly to give a clearer picture of the assembly.

Powered by Editorial Manager® and ProduXion Manager® from Aries Systems Corporation

The reviewer raises a good point, which we should have been more clear about in the text. We agree that N50/90/95 and L50/90/95 are important statistics to evaluate a genome assembly landscape. In order to keep the text concise, we have adjusted the manuscript to include both N/L50 and N/L95, but include the additional N/L90 values in the supplemental materials, given their similarity to the N/L90 values. The number of scaffolds that cover 90% of the genome is 24, which is 3 more than that found in the hg38 human assembly (L90 = 21). Regarding the L95 and N95 values, we see a similar trend: mLemCat1 L95 = 28 and N95 = 21.9 Mb; hg38 N95 = 24 and N95 = 46.7 Mb. As the Lemur catta genome is about two-thirds the size of the human genome these contiguity values are similar. Additionally, the expected haploid number of chromosomes [4] in Lemur catta is larger, 29 chromosomes expected (27 autosomal + 2 sexual (reference chromosomes)) is larger than the 22 autosomes and 2 sexual human chromosomes. We have added the following parameters in Table S1:

"Lemur catta (mLemCat1): L90 = 24, N90 = 30,322,482 bp ; L95 = 28, N95 = 21,924,082 bp, Span = 2,122,351,751 bp
Human (hg38): L90 = 21, N90 = 58,617,616 bp ; L95 = 24, N95 = 46,709,983 bp, Span = 3,209,286,105 bp"

* What other primate species genomes were recently assembled at "chromosome-level assembly" similar to this study and how the N50 of scaffolds from other recent primate genome assemblies is different (or similar) to N50 scaffold size of this assembly? Would be good to mention in the discussion section. There are a few other recent assemblies of primates in GigaScience (over the last 2 years) using similar methods.

Thank you for this suggestion. As the reviewer rightly mentions, there are other recent primate assemblies published in GigaScience that are valuable points of comparison. While searching the GigaScience website for published chromosome-level genomes from the past two years, we were able to identify three such assemblies: Ma2, Panubis1.0, and ASM756505v1. mLemcat1 has a slightly smaller scaffold N50 compared to these recently published primate genomes. However, the size of our Lemur catta genome assembly is at least 25% smaller than the other genome assemblies used for this comparison, which explains its proportionally smaller N50 value. We have added the Table S5: Comparison of scaffold N50 and assembly size of the latest primate genomes published in GigaScience to the supplementary materials and the corresponding text in the discussion.

* Repeat analysis would benefit from running 'repeat modeler' in addition to existing analysis.

As we have detailed above, we are confident in our RepeatMasker results and contend that the additional run of Repeat Modeler could lead to additional complications.

1. Sauther ML, Sussman RW, Gould L. The socioecology of the ringtailed lemur: Thirty-five years of research. Evol Anthropol. Wiley; 1999;8:120–32.
2. Ghurye J, Rhie A, Walenz BP, Schmitt A, Selvaraj S, Pop M, et al. Integrating Hi-C links with assembly graphs for chromosome-scale assembly. PLoS Comput Biol. 2019;15:e1007273.
3. Donath A, Jühling F, Al-Arab M, Bernhart SH, Reinhardt F, Stadler PF, et al. Improved annotation of protein-coding genes boundaries in metazoan mitochondrial genomes. Nucleic Acids Res. Oxford Academic; 2019;47:10543–52.
4. Cardone MF, Ventura M, Tempesta S, Rocchi M, Archidiacono N. Analysis of chromosome conservation in Lemur catta studied by chromosome paints and BAC/PAC probes. Chromosoma. 2002;111:348–56.
5. Roodgar M, Babveyh A, Nguyen LH, Zhou W, Sinha R, Lee H, et al. Chromosome-level de novo assembly of the pig-tailed macaque genome using linked-read sequencing and HiC proximity scaffolding. Gigascience. 2020;9.
6. Batra SS, Levy-Sakin M, Robinson J, Guillory J, Durinck S, Vilgalys TP, et al. Accurate assembly of the olive baboon (Papio anubis) genome using long-read and Hi-C data. Gigascience. 2020;9.
7. Wang L, Wu J, Liu X, Di D, Liang Y, Feng Y, et al. A high-quality genome assembly for the endangered golden snub-nosed monkey (Rhinopithecus roxellana). Gigascience. 2019;8.

Additional Information:	
**Question**	**Response**
Are you submitting this manuscript to a special series or article collection?	No
**Experimental design and statistics**<br><br>Full details of the experimental design and statistical methods used should be given in the Methods section, as detailed in our Minimum Standards Reporting Checklist. Information essential to interpreting the data presented should be made available in the figure legends.<br><br>Have you included all the information requested in your manuscript?	Yes
**Resources**<br><br>A description of all resources used, including antibodies, cell lines, animals and software tools, with enough information to allow them to be uniquely identified, should be included in the Methods section. Authors are strongly encouraged to cite Research Resource Identifiers (RRIDs) for antibodies, model organisms and tools, where possible.<br><br>Have you included the information requested as detailed in our Minimum Standards Reporting Checklist?	Yes
**Availability of data and materials**<br><br>All datasets and code on which the conclusions of the paper rely must be either included in your submission or deposited in publicly available repositories (where available and ethically appropriate), referencing such data using a unique identifier in the references and in the "Availability of Data and Materials" section of your manuscript.	Yes

Have you have met the above requirement as detailed in our Minimum Standards Reporting Checklist?	

Manuscript

Click here to access/download;Manuscript;LemurCattaRef_Reviewed_Manus

Data Note: A high-quality, long-read genome assembly of the endangered ring-tailed lemur (*Lemur catta*)

Marc Palmada-Flores[1,*], Joseph D. Orkin[1,2,*], Bettina Haase[3], Jacquelyn Mountcastle[3], Mads F. Bertelsen[4,5], Olivier Fedrigo[3], Lukas Kuderna[1], Erich D. Jarvis[3,5,6], Tomas Marques-Bonet[1,8,9,10]

1 Institut de Biologia Evolutiva, Universitat Pompeu Fabra - CSIC, Barcelona, Spain

2 Département d'anthropologie, Université de Montréal, Montréal, Canada

3 The Vertebrate Genomes Lab, The Rockefeller University, New York, New York, USA

4 Department of Veterinary and Animal Sciences, Faculty of Health and Medical Sciences, University of Copenhagen, Frederiksberg C, Denmark

5 Center for Zoo and Wild Animal Health, Copenhagen Zoo, Frederiksberg, Denmark

6 Howard Hughes Medical Institute, Chevy Chase, Maryland, USA

7 Laboratory of Neurogenetics of Language, The Rockefeller University, New York, United States

8 Catalan Institution of Research and Advanced Studies (ICREA), Barcelona, Spain

9 CNAG- CRG, Centre for Genomic Regulation (CRG), Barcelona Institute of Science and Technology (BIST), Barcelona, Spain

10 Institut Català de Paleontologia Miquel Crusafont, Universitat Autònoma de Barcelona, Barcelona, Spain

* These authors contributed equally

Marc Palmada-Flores [0000-0003-0246-5226];

Joseph D Orkin [0000-0001-6922-2072];

Bettina Haase [0000-0001-8945-7282];

Jacquelyn Mountcastle [0000-0003-1078-4905];

Mads F. Bertelsen [0000-0001-9201-7499];

Olivier Fedrigo [0000-0002-6450-7551];

Lukas Kuderna [0000-0002-9992-9295];

Erich D Jarvis [0000-0001-8931-5049];

Tomas Marques-Bonet [0000-0002-5597-3075]

Abstract

The ring-tailed lemur (*Lemur catta*) is a charismatic strepsirrhine primate endemic to the island of Madagascar. These lemurs are of particular interest, given their status as a flagship species and widespread publicity in popular media. Unfortunately, a recent population decline has caused the census population to fall below 2500 individuals in the wild, and their classification as an endangered species by the IUCN. As is the case for most strepsirrhine primates, only a limited amount of genomic research has been conducted on *L. catta*, in part due to the lack of genomic resources. We generated a new high-quality reference genome assembly for *L. catta* (mLemCat1) that conforms to the standards of the Vertebrate Genomes Project. This new long-read assembly is composed of PacBio continuous long reads (CLR data), Optical Mapping Bionano reads, Arima HiC data, and 10X linked-reads. The contiguity and completeness of the assembly is extremely high, with scaffold and contig N50 values of 90.982 Mbp and 10.570 Mbp, respectively. Additionally, when compared to other high-quality primate assemblies, *L. catta* has the lowest reported number of Alu elements, which results predominantly from a lack of AluS and AluY elements. mLemCat1 is an excellent genomic resource not only for the ring-tailed lemur community, but also for other members of the Lemuridae family and is the first very long read assembly for a strepsirrhine.

Context:

The strepsirrhines are a remarkably diverse radiation of primates that includes more than one quarter of all recognized primate species [1]. The vast majority of strepsirrhines (103 species) are members of the Lemuroidea, colloquially known as “lemurs”, and endemic to Madagascar. Despite their geographic isolation, the lemur radiation is exceptionally diverse, including both the smallest living primate (*Microcebus berthae*) and one of the largest (the recently extinct subfossil lemur, *Archaeoindris fontoynontyii*) [2,3]. Although lemurs are highly diverse, they are comparatively understudied relative to other primates, and ~87% of species are threatened with extinction, raising major conservation challenges [1].

Of particular interest, both ecologically and in the public imagination, are ring-tailed lemurs (*Lemur catta*, NCBI:txid9447). Ring-tailed lemurs are medium-bodied, ecologically flexible members of the Lemuridae family and the sole member of the genus *Lemur*. [4–6]. In contrast to most other Lemuridae, *L. catta* predominantly inhabit the dry and seasonal forests of southern Madagascar [7]. They consume an omnivorous diet mostly of fruit and leaves, and engage in a multi-male multi-female social structure with a polygynandrous mating system [7]. Ring-tailed lemurs are under severe conservation pressure; they are classified as Endangered by the IUCN [8], resulting primarily from deforestation, hunting, and capture for the pet trade. A recent population census has revealed a dramatic population decline with as few as 2200 individuals remaining in the wild [9]. Of further concern, the species is distributed across a highly fragmented range with only eight populations of at least 100 individuals remaining [9]. Despite this near-term population decline, a recent microsatellite analysis indicates that the genetic diversity of *L. catta* populations could be exceptionally high, with evidence of genetic isolation by distance throughout their geographic

range [6].

From a genomic perspective, relatively little is known about ring-tailed lemurs (and strepsirrhines more broadly). Genome assemblies have been published for 18 strepsirrhine species, but none of these assemblies has a contig N50 value above 1 Mb, and only three of them are above 100 kb [10]. Recently, a *Lemur catta* genome (LemCat_v1_BIUU) was assembled by the Zoonomia consortium [11], given that it is derived from Illumina short reads, its metrics and application are still limited compared to the genome quality of recent highly contiguous assemblies [12]. This general lack of genomic resources remains a considerable limitation for the comparative and population genomics of lemurs.

Here, we present a new high-quality genome assembly of *L. catta* (mLemCat1) that conforms to the standards of the Vertebrate Genomes Project (VGP). mLemCat1 was assembled with a combination of PacBio continuous long reads (CLR data), Optical Mapping Bionano reads, Arima HiC data, and 10X linked-reads. Our new assembly will allow for a deep assessment of the genome biology and conservation genomics of endangered ring-tailed lemurs. Additionally, given the paucity of high contiguity strepsirrhine assemblies, it will allow major advances in the genomics of across the Lemuridae family.

Data Description

Library preparation and sequencing

Spleen tissue was collected post-mortem from a male at the Copenhagen Zoo (Denmark) in 2015 and immediately flash-frozen (ZIMS Global Accession Number GAN: DKL15-03323). We isolated 30ug of ultra high molecular weight DNA (uHMW) from 35 mg of flash-frozen spleen tissue using

the agarose plug Bionano Genomics protocol for animal tissue (DNA isolation fibrous tissue protocol (#30071C). uHMW DNA quality was assessed by a Pulsed Field Gel assay and quantified with a Qubit 2 Fluorometer (Figure S1).

10µg of uHMW DNA was sheared using a 26G blunt end needle (PacBio protocol PN 101-181-000 Version 05). A large-insert PacBio library was prepared using the Pacific Biosciences Express Template Prep Kit v2.0 (#100-938-900) following the manufacturer protocol. The library was then size selected (>20kb) using the Sage Science BluePippin Size-Selection System. 23 PacBio 1M v3 (#101-531-000) smrtcells were sequenced on the Sequel instrument (PacBio Sequel System, RRID:SCR_017989) (sequencing kit 3.0 #101-597-800) with a 10 hours movie and 2 hours pre-extension time. Unfragmented uHMW DNA was used to generate a linked-reads library on the 10X Genomics Chromium (Genome Library Kit & Gel Bead Kit v2 PN-120258, Genome Chip Kit v2 PN-120257, i7 Multiplex Kit PN-120262). This 10X library was sequenced on an Illumina Novaseq (Illumina NovaSeq 6000 Sequencing System, RRID:SCR_016387) S4 150bp PE lane. uHMW DNA was labeled for Bionano Genomics optical mapping (BioNano Irys system, RRID:SCR_016754) using the Bionano Prep Direct Label and Stain (DLS) Protocol (30206E) and run on one Saphyr (Saphyr, RRID:SCR_017992) instrument chip flow cell. Hi-C preparation was performed by Arima Genomics using the Arima-HiC kit (P/N: A510008) and an Illumina-compatible library was generated using the KAPA Hyper Prep kit (P/N: KK8504). This library was then sequenced on an Illumina HiSeq Xten (Illumina HiSeq X Ten, RRID:SCR_016385) (150bp PE) at ~60× coverage following the manufacturer's protocols. Assuming a genome size of 3.21 Gbp from the GoaT database [13], the present genome (mLemCat1) has 86.43X of 10.28X linked-reads data, 66.68X of Arima data, 154.57X of Bionano data and 62.88X of PacBio data.

De novo assembly

The genome was assembled following the VGP standard pipeline v1.6 [12], and the specific parameter settings are available on the VGP github repository (Additional File 1). Specifically, contigs were generated using FALCON (FALCON, RRID:SCR_018804) [14] and FALCON-Unzip [15], producing primary and alternate assemblies. We used purge_dups (purge dups, RRID:SCR_021173) [16] to identify false duplications caused by regions of high-heterozygosity. Purged contigs were removed from the primary assembly and added to the alternate assembly. We then scaffolded the primary assembly using 10X linked-reads data with scaff10X 2.0 [17], Bionano optical maps with Bionano Solve v.2.1 [18], and Arima Hi-C data with Salsa 2.2 [19]. We assembled the mitochondrial genome separately using MitoVGP [20] with PacBio and 10X data. The primary scaffolds, alternate contigs, and mitochondrial assembly were polished simultaneously. We first performed Polishing and gap filling with the original PacBio data using Arrow [14], followed by two rounds of short-reads polishing using the 10X linked-reads data. Specifically, 10X data was mapped to the assembly using Longranger 2.1.3 [21] and polishing was done with FreeBayes (FreeBayes, RRID:SCR_010761) [22]. All computing was performed on the DNAnexus (DNAnexus, RRID:SCR_011884) cloud platform.

Genome Quality Assessment

Compared to the currently available short-read *Lemur catta* genome available (LemCat_v1_BIUU) [11], the new mLemCat1 assembly has higher contiguity values, fewer scaffolds, and a slightly smaller assembly size (Table 1). We generated basic continuity assembly metrics for both assemblies using QUAST V5.0.2 (QUAST, RRID:SCR_001228) [23], which are presented in Table 1. The assembly has a total scaffold size of 2.122 Gb within 141 scaffolds. The mLemCat1 contig and scaffold N50 values are 10.570 Mb and 90.982 Mb, representing 20.41 fold and 421.21 fold increases, respectively, compared to the LemCat_v1_BIUU

assembly. In comparison with the human genome assembly (*hg38), the* L95 and N95 statistics (L95=24; N95=46.710 Mb for *hg38*; L95=28; N95=21.924 Mb for *mLemCat1*) are similar, given the expected chromosomes for both (22 autosomes + 2 sexual chromosomes in human, and 27 autosomal + 2 sexual chromosomes for *Lemur catta*) [24]. Further comparison can be found in Table S1. The overall GC content of this assembly is 40.48%.

The mLemCat1 assembly has a high level of accuracy and completeness that conforms to the proposed standards of the VGP [12]. We assessed the base and structural accuracies of the assembly with Merqury V1.1, using a Meryl V1.7 database [25] based on 130.708 Gb (84X coverage) of 10x linked-reads reads. The base pair QV of the primary assembly is 44.35, which exceeds the VGP standard. The k-mer completeness is 91.45%. We classified the structural accuracy using the false duplications percentage calculated in the *false_duplications.sh* script from Merqury V1.1. The assembly is estimated to have 0.39% false duplications based on the percentage of kmers found in unexpected copy numbers.

Table 1: Genome Quality Metrics for the mLemCat1 genome assembly compared to previous assembly and standards.

QUALITY CATEGORY	QUALITY METRIC	VGP STANDARD	mLemCat1	LemCat_v1_BIUU
Continuity	# Scaffolds	-	141	575,427
	Scaffold N50	23-480 Mbp	90.982 Mbp	0.216 Mbp
	Largest scaffold	-	285.823 Mbp	2.320 Mbp
	# Contigs	-	518	580,026

	Contig N50	1-25 Mbp	10.570 Mbp	0.158 Mbp
	Largest contig	-	40.360 Mbp	1.312 Mbp
	Gaps / Gbp	75-1500	179.5	2001.3
	Span	-	2.122 Gbp	2.298 Gbp
**Structural accuracy**	False duplications	0.2-5.0%	0.39%	-
**Base accuracy**	Base pair QV	39-43	44.45	-
	K-mer completeness	87-98%	91.45%	-
**Functional completeness**	Genes (BUSCOs (S))	82-98%	88.80%	81.46%
**Chromosome status**	Organelles (e.g. MT)	1 Complete allele	1 Complete allele	-

S: single-copy genes, MT: mitochondrial; Gbp: giga base pairs; Mbp: mega base pairs; #: number.

In order to assess the functional completeness of the assembly, we recovered BUSCO genes from both mLemCat1 and the existing Illumina-based assembly (LemCat_v1_BIUU) (Figure 2). Specifically, we conducted a gene completeness assessment using BUSCO (BUSCO, RRID:SCR_015008) V4.0.6 [26], setting human as the reference species in the --augustus_species parameter, and using the primates_OrthoDB10 database, which comprises a total of 13780 genes. Of the 13780 possible BUSCOs, we identified 12138 single-copy (88.8%), 100 duplicates (0.7%), and 188 fragmented genes (1.4%) in mLemCat1, leaving 9.8% of BUSCOs

missing. In contrast, we could only recover 11132 single-copy BUSCOs (81.5%) from LemCat_v1_BIUU, with 15.3% of BUSCO genes missing.

The present assembly (*mLemCat1*) can be useful to create synteny plots between the present species and others, such as humans (Figure S2), as it has N50 statistics comparable to other high-quality primate genomes, like the pig-tailed macaque [27], olive baboon [28] and golden snub-nosed monkey [29] genome assemblies that have been recently published (Table S2).

Mitogenome of L. catta

We assembled a gapless mitochondrial genome with a span of 17086 bp using both PacBio CLR (long reads) and 10X data (short reads) using MitoVGP v2.2 with additional parameters "*-f 18000 -v LENIENT*", as described in the Additional File 2: Table S3 of the MitoVGP paper [20], and annotated the assembly using the *MITOS2 web server* [30]. With the annotation results we plotted a map of the mitochondrion with GenomeVx [31] (Figure S3). Thirteen main protein coding genes have been annotated in this new mitogenome including nad1, nad2, nad3, nad4, nad4L, nad5, nad6, cox1, cox2, cox3, atp6, atp8 and cob.

Analysis of the repeatome

To assess the structure and variety of repeat elements in the *L. catta* genome, we analyzed mLemCat1 with RepeatMasker (RepeatMasker, RRID:SCR_012954) 4.1.2-p1. Non-default settings included the use of sensitive mode, the query assumed species set to primates, nhmmscan 3.3.2 (Nov 2020), and FamDB: HMM-Dfam_3.3, without the exclusion of simple repeats. In total, 50.32% of the bases in the *L. catta* genome (mLemCat1) are masked as

interspersed repeats, including LINEs, SINEs, LTRs and DNA elements (Figure 3A, Table S3). In general terms, the portion of the genome that comprises repetitive elements is similar to that which has been reported for other high-quality catarrhine genomes [32,33], although there are fewer satellites (0.30%), simple repeats (0.68%) and low complexity elements (0.13) (Table S3).

In comparison with the previous Illumina-only assembly (LemCat_v1_BIUU) we observed minor differences in the structure and variety of repeat elements (Figure 4). The new long-read based assembly has 1.31% more interspersed repeats (50.32% vs 49.01%), and a higher percentage of sequence in each repeat subtype, except for satellites, simple repeats, low complexity elements, and ERV classes I & II. We also observed both a lower percentage of sequence and a smaller number of ALU events in mLemCat1. Additionally, the total number of masked bases is lower in the new assembly, but they represent a higher percentage of the sequence, due to mLemCat1 having a shorter span.

Alus are the most abundant repeat elements in the human genome, and differences in their rates, distribution, and proliferation could have led to distinct functional changes in multiple primate lineages [34]. Alu elements have been present since the earliest stages of primate evolution are frequently located in gene-rich regions, and may have an important role in gene regulation [35–38]. In order to compare the Alu repeat landscape of *L. catta* with those of other highly-contiguous primate assemblies, we ran RepeatMasker as above adding the *-alu* option. The genomes used for the comparison were long-read based assemblies, including human (hg38), chimpanzee (panTro6), western gorilla (gorGor6), Sumatran orangutan (ponAbe3), rhesus macaque (rheMac10), common marmoset (calJac4), and gray mouse lemur (Mmur_3.0) (Table S4).

We identified substantially fewer Alu elements in the lemur genomes (*L. catta* and *Microcebus murinus*) than those of the catarrhines, with the fewest being found in the *L. catta* genome (3.66%

of repeat elements) (Figure 3B, Table S5). In contrast to the other primates assessed, for which AluS elements are most abundant, AluJ is the most common element in mLemCat1 (54.17% of Alu events). Both lemurs have fewer AluS events than the anthropoids and fewer AluY events than the catarrhines, consistent with previous reports of the expansion of these two families after the Catarrhini-Strepsirrhini split [39]. The fact that the common marmoset has the highest number of AluS elements (Figure 3C) confirms that the burst that started before the Catarrhini and Platyrrhini parvorders diverged, continued with different activity in both lineages after their split. Recent Alu activity (AluY events), is most abundant in catarrhines, particularly the rhesus macaque, which when compared to great apes (Figure 3C), has a higher overall percentage of Alus (Figure 3B).

CONCLUSION

We have assembled a new high-quality genome reference for the ring-tailed lemur (*L. catta*) that satisfies the VGP quality assembly standards. Compared to pre-existing genomic resources, the new assembly has higher contiguity and completeness, and contains more single copy complete BUSCO genes with fewer fragmented or missing genes. Additionally, we analyzed the *L. catta* repeatome and observed substantially fewer Alu events compared to other high-quality primate assemblies. This assembly illustrates how long-reads and further scaffolding data such as HiC or optical mappings can drastically improve the contiguity and completeness of an assembly, which also allows for improved analysis of structural variation. We suggest that this new assembly will be an excellent resource for the mammalian genomics community, with particular value for the conservation genomics of lemurs.

DATA AVAILABILITY

The raw sequencing data and assembly are available via NCBI BioProject: PRJNA562215. mLemCat1 assembly and the raw reads used to generate it can be accessed at GenomeArk https://vgp.github.io/genomeark/Lemur_catta/. The complete mitogenome of mLemCat1 is available in Genomeark as mLemCat1.MT.20190820.fasta.gz https://vgp.github.io/genomeark/Lemur_catta/. Specific command line parameters are available in Additional File 1. The supporting datasets are available in the *GigaScience* database (GigaDB) [40].

ADDITIONAL FILE

Additional file 1: Links to the websites with the assembly pipeline specifics used to create *Lemur catta* (mLemCat1) genome assembly and command lines used to perform the different analyses.

AUTHOR CONTRIBUTIONS

MPF and JDO analyzed the data; JM and BH generated the data; BH generated the draft assembly; MFB collected the samples. MPF and JDO wrote the paper with contributions from all authors. TMB, EFJ, and OF designed the research.

ACKNOWLEDGEMENTS

The project that gave rise to these results received the support of two fellowship from ”la Caixa” Foundation (ID 100010434) and from the European Union’s Horizon 2020 research and innova-

tion programme under the Marie Skłodowska-Curie grant agreement No 847648. The fellow- ship codes are LCF/BQ/PI20/11760004 & LCF/BQ/DR20/11790032. TMB is supported by funding from the European Research Council (ERC) under the European Union's Horizon 2020 research and innovation programme (grant agreement No. 864203), BFU2017-86471-P (MINECO/FEDER, UE), "Unidad de Excelencia María de Maeztu", funded by the AEI (CEX2018-000792-M), Howard Hughes International Early Career, NIH 1R01HG010898-01A1 and Secretaria d'Universitats i Recerca and CERCA Programme del Departament d'Economia i Coneixement de la Generalitat de Catalunya (GRC 2017 SGR 880).

REFERENCES

1. Estrada A, Garber PA, Rylands AB, Roos C, Fernandez-Duque E, Di Fiore A, et al. Impending extinction crisis of the world's primates: Why primates matter. Sci Adv. 2017;3:e1600946.

2. Orkin JD, Kuderna LFK, Marques-Bonet T. The Diversity of Primates: From Biomedicine to Conservation Genomics. Annu Rev Anim Biosci. 2021;9:103–24.

3. Fleagle JG. Primate Adaptation and Evolution. Academic Press; 2013. https://doi.org/10.1016/C2009-0-01979-5

4. Sussman RW. Demography and social organization of free-ranging *Lemur catta* in the Beza Mahafaly Reserve, Madagascar. Am J Phys Anthropol. Wiley; 1991;84:43–58.

5. Cameron A, Gould L. Fragment-Adaptive Behavioural Strategies and Intersite Variation in the Ring-Tailed Lemur (*Lemur catta*) in South-Central Madagascar. In: Marsh LK, Chapman CA, editors. Primates in Fragments: Complexity and Resilience. New York, NY: Springer New York; 2013. p. 227–43.

6. Chandrashekar A, Knierim JA, Khan S, Raboin DL, Venkatesh S, Clarke TA, et al. Genetic

population structure of endangered ring-tailed lemurs (*Lemur catta*) from nine sites in southern Madagascar. Ecol Evol. 2020;10:8030–43.

7. Sauther ML, Sussman RW, Gould L. The socioecology of the ringtailed lemur: Thirty-five years of research. Evol Anthropol. Wiley; 1999;8:120–32.

8. Lafleur M, Gould L. *Lemur catta*. The IUCN Red List of Threatened Species. 2020;e.T11496A115565760. http://dx.doi.org/10.2305/IUCN.UK.2020-2.RLTS.T11496A115565760.en.

9. LaFleur M, Clarke TA, Reuter K, Schaeffer T. Rapid Decrease in Populations of Wild Ring-Tailed Lemurs (*Lemur catta*) in Madagascar. Folia Primatol. 2016;87:320–30.

10. Kuderna LF, Esteller-Cucala P, Marques-Bonet T. Branching out: what omics can tell us about primate evolution. Curr Opin Genet Dev. 2020;62:65–71.

11. Zoonomia Consortium. A comparative genomics multitool for scientific discovery and conservation. Nature. 2020;587:240–5.

12. Rhie A, McCarthy SA, Fedrigo O, Damas J, Formenti G, Koren S, et al. Towards complete and error-free genome assemblies of all vertebrate species. Nature. Nature Publishing Group; 2021;592:737–46.

13. Challis RJ, Kumar S, Stevens L, Blaxter M. GenomeHubs: simple containerized setup of a custom Ensembl database and web server for any species. Database. 2017;2017. http://dx.doi.org/10.1093/database/bax039

14. Chin C-S, Alexander DH, Marks P, Klammer AA, Drake J, Heiner C, et al. Nonhybrid, finished microbial genome assemblies from long-read SMRT sequencing data. Nat Methods. 2013;10:563–9.

15. Chin C-S, Peluso P, Sedlazeck FJ, Nattestad M, Concepcion GT, Clum A, et al. Phased diploid genome assembly with single-molecule real-time sequencing. Nat Methods. 2016;13:1050–4.

16. Guan D, McCarthy SA, Wood J, Howe K, Wang Y, Durbin R. Identifying and removing haplotypic duplication in primary genome assemblies. Bioinformatics. 2020;36:2896–8.

17. Ning Z, Harry E. Scaff10X. 2021. https://github.com/wtsi-hpag/Scaff10X

18. Bionano Genomics, Inc. Bionano Software Downloads. 2021. https://bionanogenomics.com/support/software-downloads/

19. Ghurye J, Rhie A, Walenz BP, Schmitt A, Selvaraj S, Pop M, et al. Integrating Hi-C links with assembly graphs for chromosome-scale assembly. PLoS Comput Biol. 2019;15:e1007273.

20. Formenti G, Rhie A, Balacco J, Haase B, Mountcastle J, Fedrigo O, et al. Complete vertebrate mitogenomes reveal widespread repeats and gene duplications. Genome Biology. 2021;22:120.

21. Bishara A, Liu Y, Weng Z, Kashef-Haghighi D, Newburger DE, West R, et al. Read clouds uncover variation in complex regions of the human genome. Genome Res. 2015;25:1570–80.

22. Garrison E, Marth G. Haplotype-based variant detection from short-read sequencing. arXiv [q-bio.GN]. 2012. http://arxiv.org/abs/1207.3907

23. Gurevich A, Saveliev V, Vyahhi N, Tesler G. QUAST: quality assessment tool for genome assemblies. Bioinformatics. 2013;29:1072–5.

24. Cardone MF, Ventura M, Tempesta S, Rocchi M, Archidiacono N. Analysis of chromosome conservation in *Lemur catta* studied by chromosome paints and BAC/PAC probes. Chromosoma. 2002;111:348–56.

25. Rhie A, Walenz BP, Koren S, Phillippy AM. Merqury: reference-free quality, completeness, and phasing assessment for genome assemblies. Genome Biol. 2020;21:245.

26. Simão FA, Waterhouse RM, Ioannidis P, Kriventseva EV, Zdobnov EM. BUSCO: assessing genome assembly and annotation completeness with single-copy orthologs. Bioinformatics. 2015;31:3210–2.

27. Roodgar M, Babveyh A, Nguyen LH, Zhou W, Sinha R, Lee H, et al. Chromosome-level de novo assembly of the pig-tailed macaque genome using linked-read sequencing and HiC proximity scaffolding. Gigascience. 2020;9:7.

28. Batra SS, Levy-Sakin M, Robinson J, Guillory J, Durinck S, Vilgalys TP, et al. Accurate assembly of the olive baboon (*Papio anubis*) genome using long-read and Hi-C data. Gigascience. 2020;9.

29. Wang L, Wu J, Liu X, Di D, Liang Y, Feng Y, et al. A high-quality genome assembly for the endangered golden snub-nosed monkey (*Rhinopithecus roxellana*). Gigascience. 2019;8.

30. Donath A, Jühling F, Al-Arab M, Bernhart SH, Reinhardt F, Stadler PF, et al. Improved annotation of protein-coding genes boundaries in metazoan mitochondrial genomes. Nucleic Acids Res. Oxford Academic; 2019;47:10543–52.

31. Conant GC, Wolfe KH. GenomeVx: simple web-based creation of editable circular chromosome maps. Bioinformatics. 2008;

32. He Y, Luo X, Zhou B, Hu T, Meng X, Audano PA, et al. Long-read assembly of the Chinese rhesus macaque genome and identification of ape-specific structural variants. Nat Commun. 2019;10:1–14.

33. Kronenberg ZN, Fiddes IT, Gordon D, Murali S, Cantsilieris S, Meyerson OS, et al. High-

resolution comparative analysis of great ape genomes. Science. 2018;360:eaar6343.

34. Batzer MA, Deininger PL, Hellmann-Blumberg U, Jurka J, Labuda D, Rubin CM, et al. Standardized nomenclature for Alu repeats. J Mol Evol. 1996;42:3–6.

35. Liu GE, Alkan C, Jiang L, Zhao S, Eichler EE. Comparative analysis of Alu repeats in primate genomes. Genome Res. genome.cshlp.org; 2009;19:876–85.

36. Urrutia AO, Ocaña LB, Hurst LD. Do Alu repeats drive the evolution of the primate transcriptome? Genome Biol. 2008;9:R25.

37. Oliver KR, Greene WK. Mobile DNA and the TE-Thrust hypothesis: supporting evidence from the primates. Mob DNA. 2011;2:8.

38. Kurosaki T, Ueda S, Ishida T, Abe K, Ohno K, Matsuura T. The Unstable CCTG Repeat Responsible for Myotonic Dystrophy Type 2 Originates from an AluSx Element Insertion into an Early Primate Genome. PLoS One. 2012;7:e38379.

39. Konkel MK, Walker JA, Batzer MA. LINEs and SINEs of primate evolution. Evol Anthropol. 2010;19:236–49.

40. Palmada-Flores M; Orkin JD; Haase B; Mountcastle J; Bertelsen MF; Fedrigo O, et al. Supporting data for "A high-quality, long-read genome assembly of the endangered ring-tailed lemur (Lemur catta)" GigaScience Database. 2022; http://dx.doi.org/10.5524/102199.

FIGURE LEGENDS

Figure 1: Ring-tailed lemur (*L. catta*); photo courtesy of Copenhagen Zoo

Figure 2: BUSCO Assessment Results comparison between mLemCat1 and LemCat_v1_BIUU *Lemur catta* assemblies using the Primates_ODB10 database (n = 13780). The new mLemCat1 assembly shows a 7.3% increase in complete single copy orthologous genes.

Figure 3: A) Percentages of elements in the *L. catta* genome (mLemCat1) masked by RepeatMasker. B) Percentage of Alus masked in primate long-read assemblies. C) Spider plots of the total number of different Alu-like elements masked in each genome assembly. Lemurs have fewer AluS elements than anthropoid primates. Axis values represent 1,000x events. FAM (Fossil Alu Monomer); FLAMs (Free Left Alu Monomers); FRAMs (Free Right Alu Monomers); AluJ (oldest); AluS (intermediate); AluY (youngest); Alu (non-specified) [31].

Figure 4: Comparison of repeat variety and structure between mLemCat1 and LemCat_V1_BIUU assemblies

Figure1_Lemurcatta

Click here to access/download;Figure;Figure1_Lemurcatta.pdf

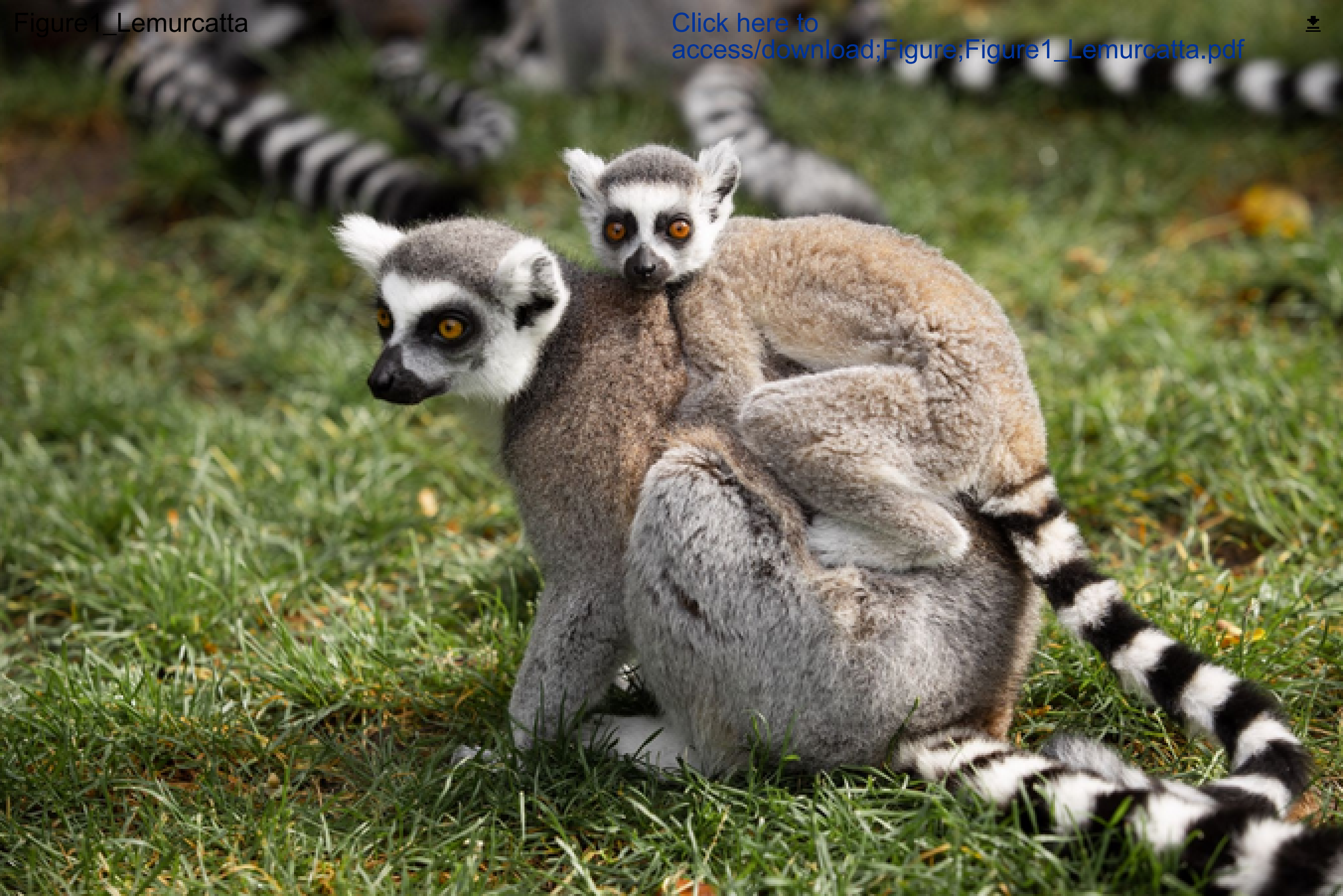

Figure2_BUSCOs

Click here to access/download;Figure;Figure2_BUSCOs.pdf

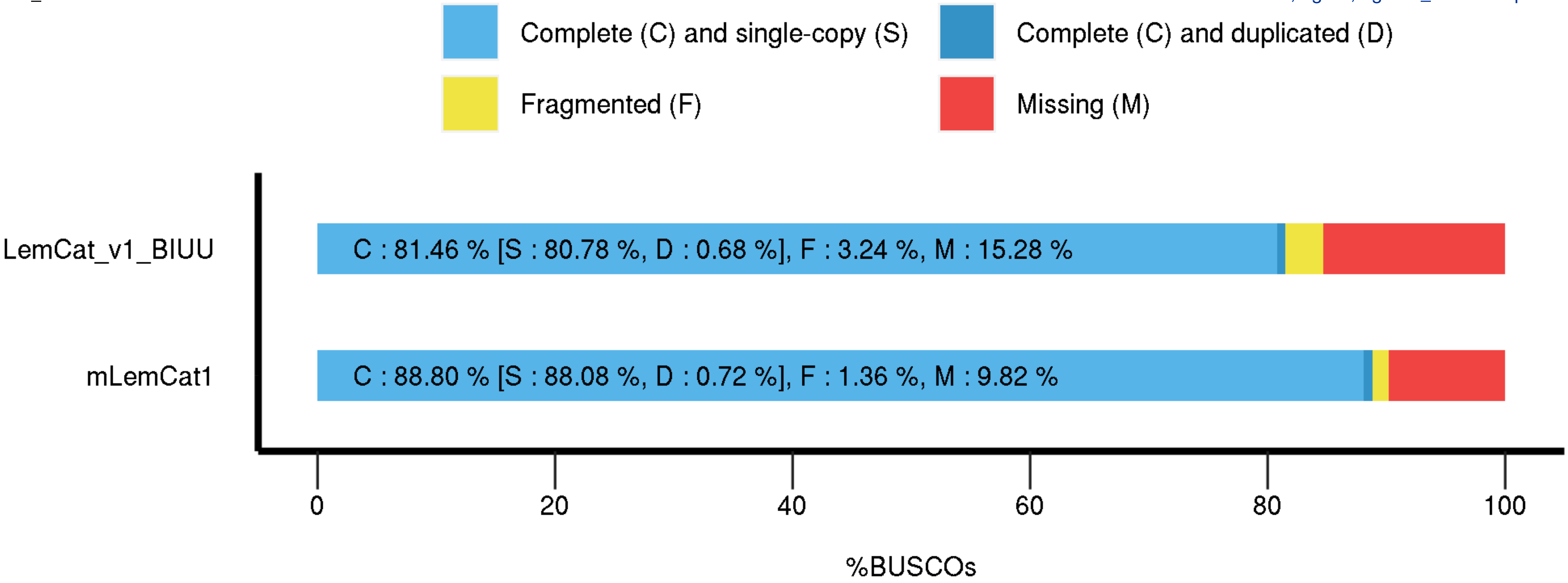

Figure3_Repeats

Click here to access/download;Figure;Figure3_Repeats.pdf

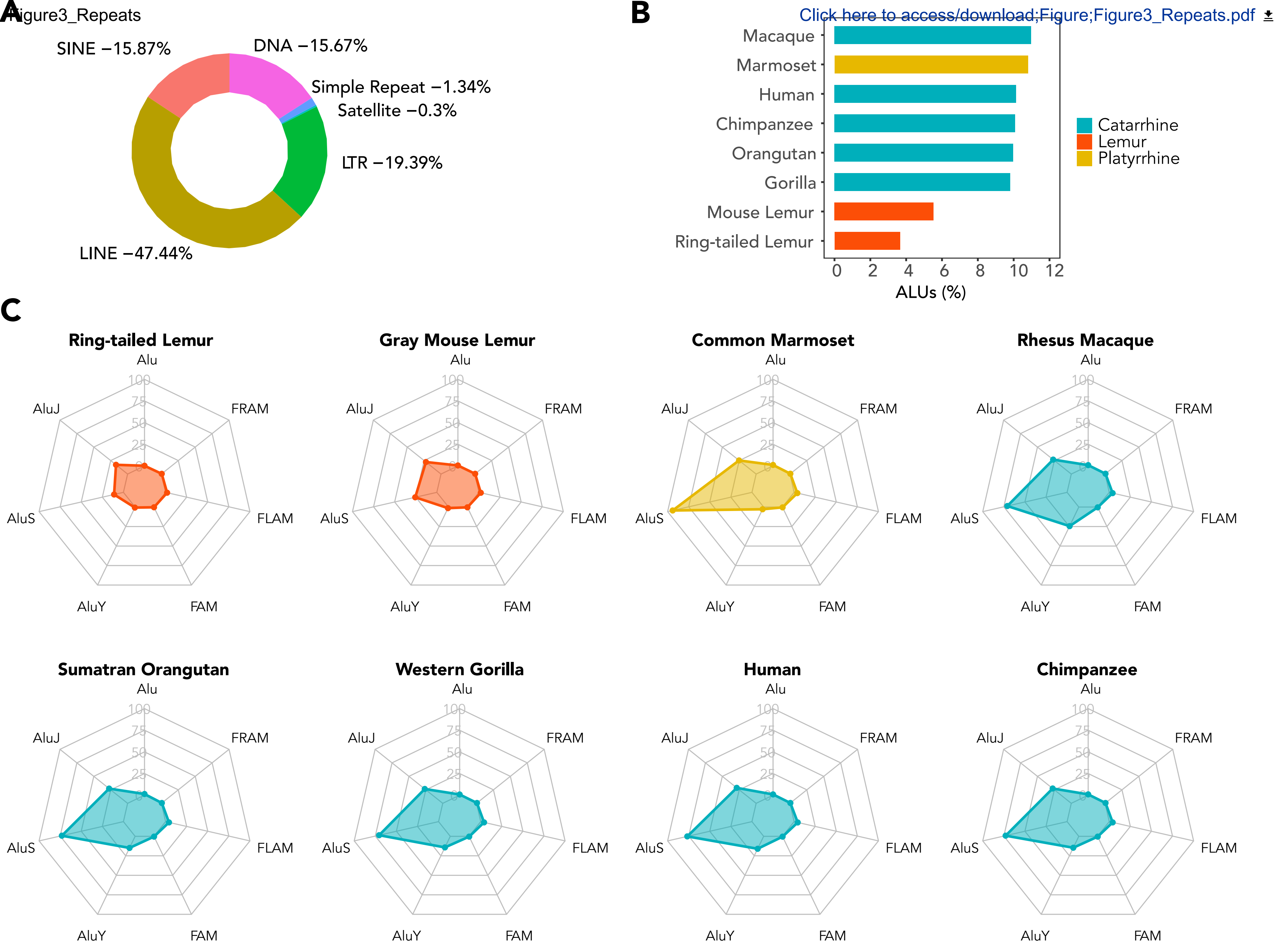

Click here to access/download;Figure;Figure4_RepeatComparisonPlot.pd

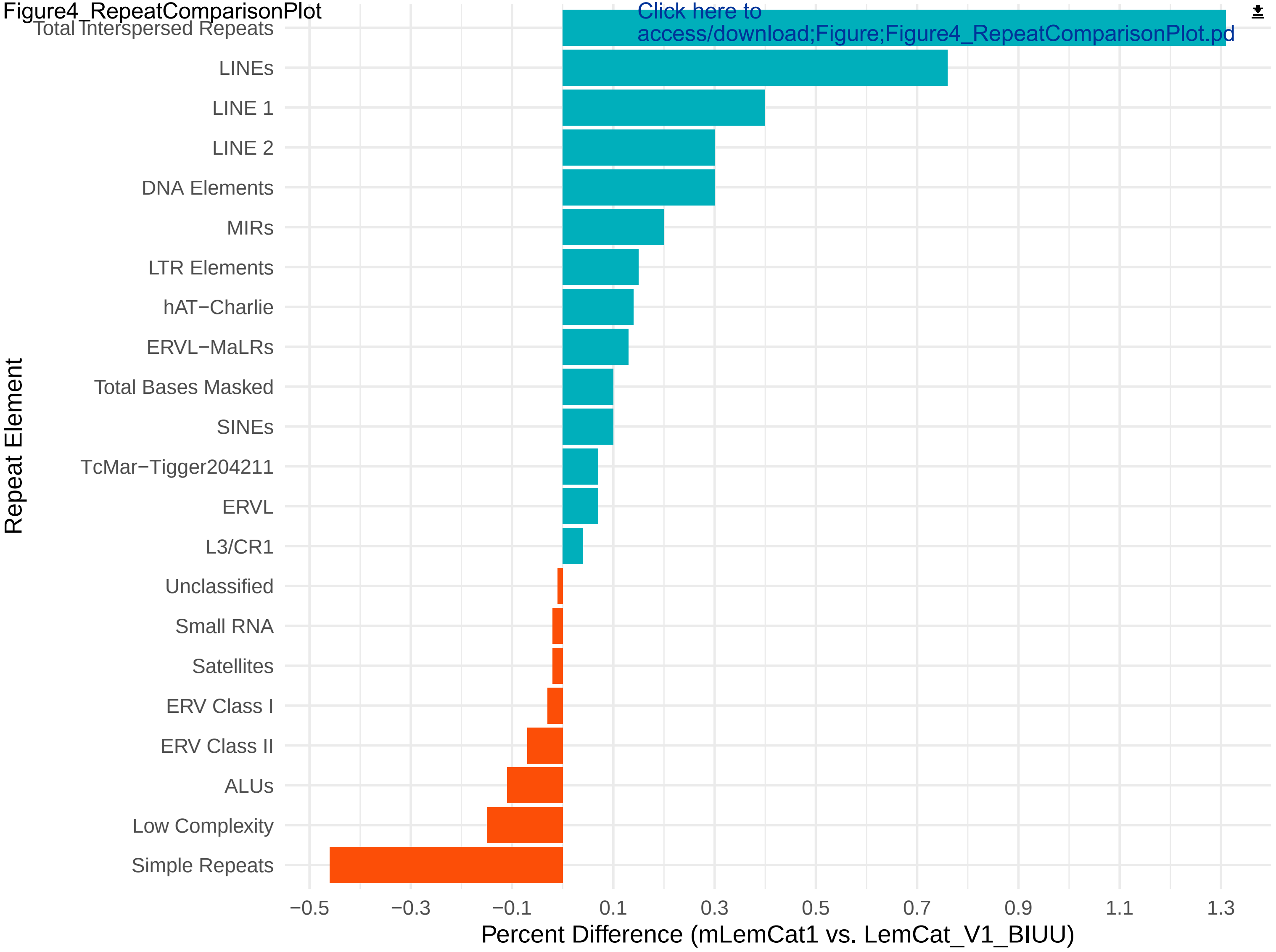

Click here to access/download
Supplementary Material
LemurCattaRef_Supplementary_Material_18022022.doc
x

Additional_File_1

Click here to access/download
Supplementary Material
Additional_File_1.docx

Cover_letter_LemurCattaRef_revision
Click here to access/download;Personal Cover;Cover_letter_LemurCattaRef_revision.docx

upf. Universitat Pompeu Fabra Barcelona

January 14th, 2022

Dear GigaScience Editorial Board,

We are pleased to submit a revised version of our manuscript, “A high-quality, long-read genome assembly of the endangered ring-tailed lemur (*Lemur catta*)” by Marc Palmada-Flores *et al*. We are grateful for the opportunity to revise our manuscript and feel it has been much improved by the valuable and constructive feedback from the reviewers and editor. We hope our manuscript is now found to be acceptable for publication in GigaScience. We include a detailed response to the editor and reviewers and a clean version of our manuscript for review.

Following the suggestions of the reviewers, we have made the following revisions to our manuscript. First, the main text now includes a paragraph supporting the comparative power of this assembly, more specific descriptions of the methodology that we used for genome and mitogenome assembly, and further description of *Lemur catta* behavioral ecology. Secondly, we added two supplementary figures: a gel illustrating the extraction process (Figure S1) and a synteny plot comparing our *Lemur catta* assembly to the human hg38 assembly (Figure S2). We also added two supplementary tables (Tables S1 and S2) describing the quality metrics of mLemCat1 and comparing them to those of human and other recently published high-quality primate assemblies, respectively. Finally, we included an Additional File with links to the websites with the assembly pipeline specifics and command lines used to perform the different analyses. We hope that these changes are satisfactory, and we look forward to your response.

We assert that this work has been approved by all authors, has not been submitted elsewhere for publication, and declare no conflicts of interest.

Thank you for considering our manuscript.

Sincerely,

Marc Palmada-Flores
Joseph D. Orkin
Tomas Marques-Bonet
