# Supplementary material for: A high-quality, long-read genome assembly of the endangered ring-tailed lemur (Lemur catta)
Source: Gigascience. 2022 Apr 1;11:giac026. doi: 10.1093/gigascience/giac026 (PMC8975718; doi:10.1093/gigascience/giac026)
Supplement: giac026_Supplemental_Files [file giac026_supplemental_files.zip › Additional_File_1.docx]

**Additional File 1 for Data Note: A high-quality, long-read genome assembly of the endangered ring-tailed lemur (*Lemur catta*)**

**Links to the websites with the assembly pipeline specifics used to create *Lemur catta* mLemCat1 genome assembly:**

Pipeline used to assemble the *Lemur catta* (mLemCat1) genome assembly:

<https://github.com/VGP/vgp-assembly/tree/master/pipeline>

Falcon unzip parameters can be found here:

<https://github.com/VGP/vgp-assembly/blob/master/dx_workflows/vgp_falcon_and_unzip_assembly_workflow/dxworkflow.json>

10X scaffolding step script used:

<https://github.com/VGP/vgp-assembly/blob/master/pipeline/scaff10x/scaff10x.sh>

Bionano scaffolding step the config.xml file:

<https://github.com/VGP/vgp-assembly/blob/master/pipeline/bionano/hybridScaffold_DLE1_config.xml>

Salsa scaffolding step script used:

<https://github.com/VGP/vgp-assembly/blob/master/pipeline/salsa/salsa2.2.sh>

**Command lines used to perform the different analyses:**

BUSCOs command line used with the lineage set as primates_OrthoDB10:

busco --offline -c ${THREADS} -i ${assembly} -l ${LINEAGE} -m genome --augustus_species human -o ${OUT}

RepeatMasker command line used to mask for all repeats:

/apps/REPEATMASKER/4.1.2/RepeatMasker -pa ${cpus} -dir ${outdir} -s -species primates -noisy ${assembly}

RepeatMasker command line used to mask ALUs:

apps/REPEATMASKER/4.1.2/RepeatMasker -pa ${cpus} -dir ${outdir} -alu -s -species primates -noisy ${assembly}
