# Supplementary material for: A high-quality, long-read genome assembly of the endangered ring-tailed lemur (Lemur catta)
Source: Gigascience. 2022 Apr 1;11:giac026. doi: 10.1093/gigascience/giac026 (PMC8975718; doi:10.1093/gigascience/giac026)
Supplement: giac026_Supplemental_Files [file giac026_supplemental_files.zip › LemurCattaRef_Supplementary_Material_18022022.docx]

**SUPPLEMENTARY MATERIAL**

**Data Note: A high-quality, long-read genome assembly of the endangered ring-tailed lemur (*Lemur catta*)**

Marc Palmada-Flores^1,*^, Joseph D. Orkin^1,2,*^, Bettina Haase^3^, Jacquelyn Mountcastle^3^, Mads F. Bertelsen^4,5^, Olivier Fedrigo^3^, Lukas Kuderna^1^, Erich D. Jarvis^3,5,6^, Tomas Marques-Bonet^1,8,9,10^

**Table S1:** Continuity metrics of human (hg38) and *Lemur catta* (mLemCat1) assemblies

| **Continuity metric** | **mLemCat1 (*Lemur catta*)** | **hg38 (*Homo sapiens*)** |
| --- | --- | --- |
| **Scaffold L50** | 9 | 9 |
| **Scaffold N50 (bp)** | 93,279,816 | 145,138,636 |
| **Scaffold L90** | 24 | 21 |
| **Scaffold N90 (bp)** | 30,322,482 | 58,617,616 |
| **Scaffold L95** | 28 | 24 |
| **Scaffold N95** **(bp)** | 21,924,082 | 46,709,983 |
| **Span (Gb)** | 2,122,351,751 | 3,209,286,105 |

**Table S2:** Comparison of scaffold N50 and assembly size of the latest primate genomes published in GigaScience

| **QUALITY CATEGORY** | **QUALITY METRIC** | **mLemCat1 (*Lemur catta*)** | **Ma2 (*Macaca nemestrina*)** | **Panubis1.0**  **(*Papio anubis*)** | **ASM756505v1 (*Rhinopithecus roxellana*)** |
| --- | --- | --- | --- | --- | --- |
| **Continuity** | Scaffold N50 (Mbp) | 91.0 | 127.5 | 140.3 | 144.6 |
|  | Span (Gbp) | 2.12 | 2.92 | 2.87 | 3.04 |
| **Reference** | | This study | [[27]](https://paperpile.com/c/uxEEhf/MSoK) | [[28]](https://paperpile.com/c/uxEEhf/bvkj) | [[29]](https://paperpile.com/c/uxEEhf/wVoa) |

**Table S3**: Repetitive elements identified by RepeatMasker in the *L. catta* genome assembly (mLemCat1)

| **Repetitive element** | | **Number of elements*** | **Length occupied (bp)** | **Percentage of the assembly (%)** |
| --- | --- | --- | --- | --- |
| SINEs: | | 975773 | 171088228 | 8.06 |
|  | ALUs | 303773 | 77664585 | 3.66 |
|  | MIRs | 656039 | 91335591 | 4.30 |
| LINEs: | | 1140393 | 511474252 | 24.10 |
|  | LINE1 | 595053 | 359987847 | 16.96 |
|  | LINE2 | 465779 | 129265242 | 6.09 |
|  | L3/CR1 | 59050 | 14804438 | 0.70 |
| LTR elements: |  | 872104 | 209147736 | 9.85 |
|  | ERVL | 212537 | 55382958 | 2.61 |
|  | ERVL-MaLRs | 252313 | 78358817 | 3.69 |
|  | ERV_classI | 251370 | 47454116 | 2.24 |
|  | ERV_classII | 22361 | 2590738 | 0.12 |
| DNA elements: | | 1169425 | 169044000 | 7.96 |
|  | hAT-Charlie | 543135 | 72642558 | 3.42 |
|  | TcMar-Tigger | 187193 | 44370166 | 2.09 |
| Unclassified: | | 105680 | 7178032 | 0.34 |
| Total interspersed repeats: | |  | 1067932248 | 50.32 |
| Small RNA: | | 129977 | 6609680 | 0.31 |
| Satellites: | | 60643 | 3268960 | 0.15 |
| Simple repeats: | | 322577 | 14421376 | 0.68 |
| Low complexity: | | 51933 | 2682469 | 0.13 |

Most repeats fragmented by insertions or deletions have been counted as single elements.

**Table S4**: Primate assembly lengths and respective quantifications of their Alu content

| Genome ID | hg38 | panTro6 | gorGor6 | ponAbe3 | rheMac10 | calJac4 | Mmur_3.0 | mLemCat1 |
| --- | --- | --- | --- | --- | --- | --- | --- | --- |
| Species | *Homo sapiens* | *Pan troglodytes* | *Gorilla gorilla* | *Pongo abelii* | *Macaca mulatta* | *Callithrix jacchus* | *Microcebus murinus* | *Lemur catta* |
| Assembly span (Gb) | 3,21 | 3,05 | 3,04 | 3,07 | 2,97 | 2,90 | 2,49 | 2,12 |
| Assembly span excluding Ns (Mb) | 3049,32 | 3018,59 | 2999,03 | 3043,44 | 2936,89 | 2859,82 | 2386,35 | 2105,70 |
| Alu elements counts | 1234675 | 1171324 | 1133522 | 1178114 | 1236178 | 1234909 | 504507 | 301914 |
| Alu total length (Mb) | 325,00 | 307,54 | 298,67 | 305,62 | 326,12 | 313,01 | 132,97 | 77,77 |
| Portion of the assembly masked as Alu element (%) | 10,13 | 10,08 | 9,81 | 9,97 | 10,98 | 10,80 | 5,52 | 3,66 |
| Alu count/Mb | 404,90 | 388,04 | 377,96 | 387,10 | 420,91 | 431,81 | 211,41 | 143,38 |

**Table S5**: Alu-monomers and Alu subfamilies count in each assembly

| Assembly ID | Species | FAM | FRAM | FLAM | AluJ | AluS | AluY | Alu | Total elements |
| --- | --- | --- | --- | --- | --- | --- | --- | --- | --- |
| hg38 | *Homo sapiens* | 4168 | 6441 | 42449 | 286811 | 766701 | 159853 | 9069 | 1275492 |
| panTro6 | *Pan troglodytes* | 3910 | 6254 | 40408 | 274933 | 728711 | 146542 | 9284 | 1210042 |
| gorGor6 | *Gorilla gorilla* | 3896 | 6139 | 39402 | 265928 | 704610 | 142210 | 7964 | 1170149 |
| ponAbe3 | *Pongo abelii* | 3895 | 6182 | 40289 | 273303 | 729945 | 149054 | 13681 | 1216349 |
| rheMac10 | *Macaca mulatta* | 3919 | 6110 | 39606 | 267762 | 708793 | 244682 | 9091 | 1279963 |
| calJac4 | *Callithrix jacchus* | 4143 | 5815 | 38049 | 252655 | 940025 | 28283 | 11194 | 1280164 |
| Mmur_3.0 | *Microcebus murinus* | 3549 | 5079 | 21194 | 222843 | 255932 | 13934 | 5221 | 527752 |
| mLemCat1 | *Lemur catta* | 2996 | 4754 | 17997 | 171397 | 110563 | 6967 | 1827 | 316501 |


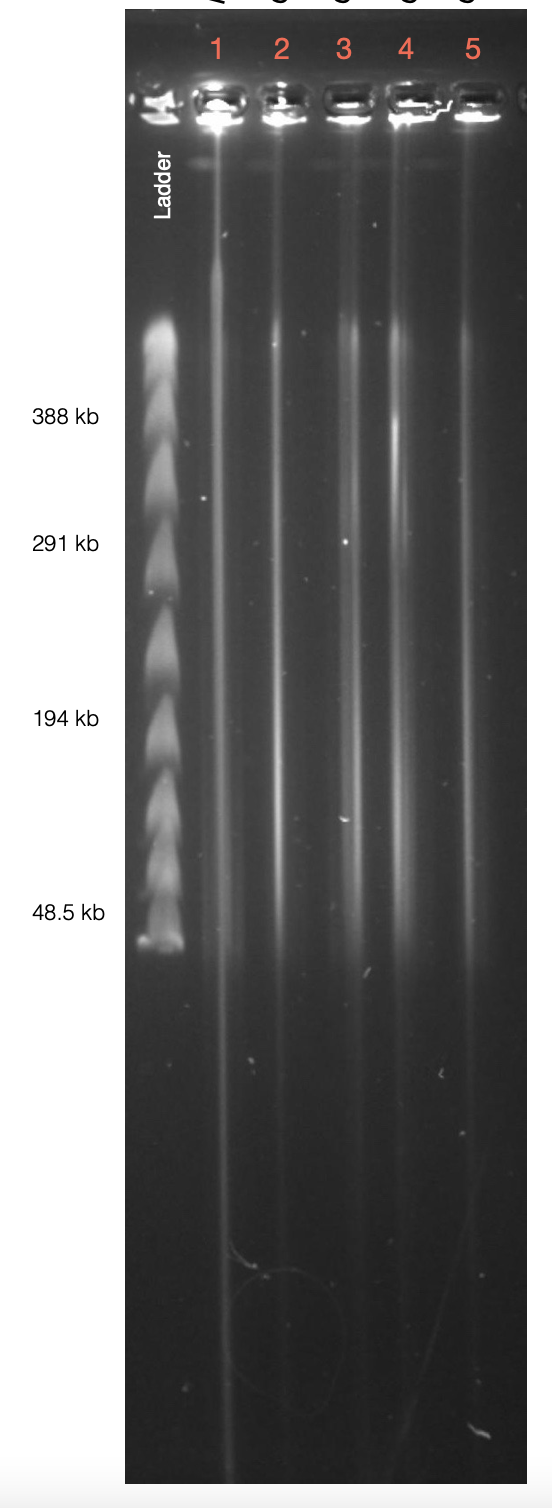


**Figure S1**: Pulse Field Gel assay (Sage Pippin Pulse) used for quality control of the ultra-High Molecular Weight DNA (*Lemur catta* is on well number 1)


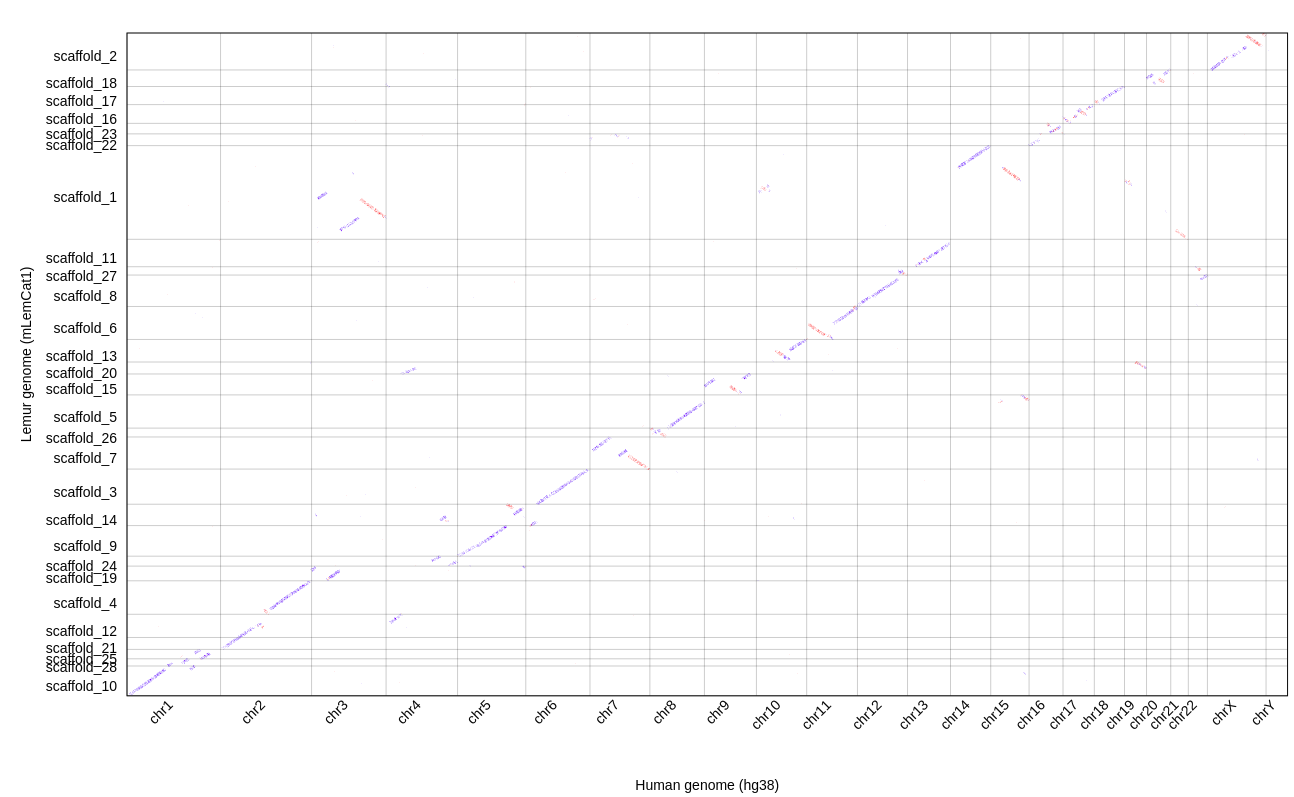


**Figure S2**: A chromosomal overall synteny plot between *Lemur catta* (mLemCat1 assembly; vertical axis) and *Homo sapiens* (hg38 assembly; horizontal axis)


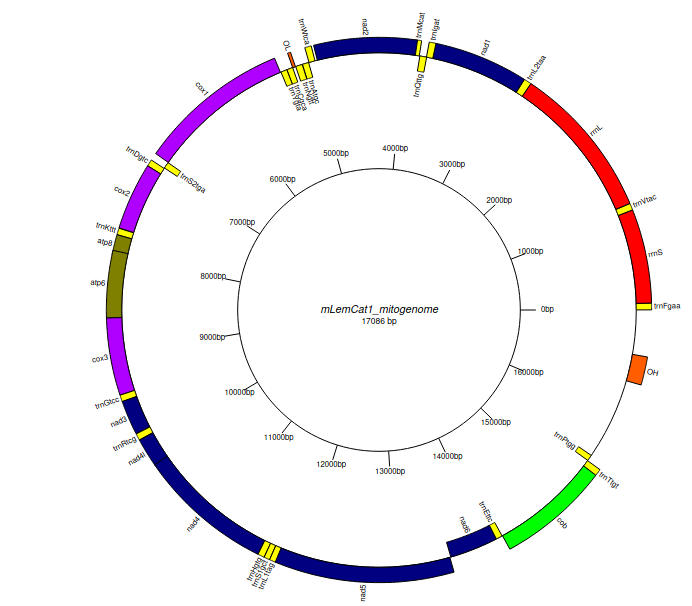


**Figure S3**: Representation of the *Lemur catta* mitogenome (mLemCat1.MT.20190820). Any genomic element annotated in the direct strand is represented in the outer layer and the ones present in the reverse strand in the inner layer.
