# Supplementary material for: A high-quality, long-read genome assembly of the endangered ring-tailed lemur (Lemur catta)
Source: Gigascience. 2022 Apr 1;11:giac026. doi: 10.1093/gigascience/giac026 (PMC8975718; doi:10.1093/gigascience/giac026)
Supplement: giac026_Response_to_Reviewer_Comments_Revision_1 [file giac026_response_to_reviewer_comments_revision_1.pdf]

Author's Response To Reviewer Comments

Close

GIGA-D-21-00335
Data Note: A high-quality, long-read genome assembly of the endangered ring-tailed lemur (Lemur catta)
Marc Palmada-Flores; Joseph D. Orkin; Bettina Haase; Jacquelyn Mountcastle; Mads F. Bertelsen; Olivier Fedrigo; Lukas Kuderna; Erich D. Jarvis; Tomas Marques-Bonet
GigaScience

****

Response to Editor:

Your manuscript "Data Note: A high-quality, long-read genome assembly of the endangered ring-tailed lemur (Lemur catta)" (GIGA-D-21-00335) has been assessed by our reviewers. Based on these reports, and my own assessment as Editor, I am pleased to inform you that it is potentially acceptable for publication in GigaScience, once you have carried out some essential revisions suggested by our reviewers.

We thank the Editor for inviting a resubmission and for the supportive words about our study and our approach. Below, we detail how we have responded to each of the constructive points raised by the reviewers. We believe our manuscript is improved through addressing these numerous helpful points and we now hope you find it suitable for publication in GigaScience.

****

Response to Reviewers:

Reviewer #1: The manuscript of "A high-quality, long-read genome assembly of the endangered ring-tailed lemur (Lemur catta)" reports a updated genome assembly for ring-tailed lemur (Lemur catta), a Strepsirrhine primate species. In combination with PacBio continuous long reads (CLR reads), Bionano reads, HiC data, and 10X linked-reads, the contig and scaffold N50 in the newly acquired genome assembly each reached to 10.570 Mbp and 90.982 Mbp. This genome assembly statistic represents 20.41 fold and 421.21 fold increases, respectively, which high quality reference genome could be served as a valuable data resource compared with the previous short-read genome of the species. As the first reported long read assembly for a Lemuriformes, one infraorder within Strepsirrhine, this genomic resource distinguished with previous report which typically focused on higher-primate, especially the apes and old-world monkeys. The release of this genome could potentially facilitate further comparable genomic analysis, help on the understanding of adaptive evolution in primates from Strepsirrhine to Haplorrhini. This updated genome is expected to gain more attention in the research areas of comparative genomics, genetics, conservation and behavior in primates as well as mammals.
The manuscript is well written, technically correct. I suggest accept this paper after minor revision.

Some questions belowing may be helpful to improve the manuscript.

We are very grateful to the reviewer for the positive assessment of our manuscript and welcome the suggestion of acceptance after minor revisions. Please take note of our responses to the specific questions below.

1. In the introduction section, beside background of distribution and taxonomy of ring-tailed lemurs, more information will be appreciate including phylogeny position and their biological background such as diet, behavior on so on.

Thank you for this suggestion. We have extended the introductory paragraph to include the following

text about ring-tailed lemur ecology and phylogenetic positioning.

“Ring-tailed lemurs are medium-bodied, ecologically flexible members of the Lemuridae family and the sole member of the genus Lemur. In contrast to most other Lemuridae, L. catta predominantly inhabit the dry and seasonal forests of southern Madagascar [1]. They consume an omnivorous diet mostly of fruit and leaves, and engage in a multi-male multi-female social structure with a polygynandrous mating system [1].”

2. During the de novo assembly and subsequent analysis, the authors use several different software packages for their analysis. However, the specific parameter settings for the software used were not given.

Thank you for drawing our attention to this issue, which we have now clarified in the text and added in Additional File 1. In order to keep the text concise, we had not listed every parameter and setting explicitly in the text. However, we have now included a link to the VGP master pipeline in the “De novo assembly” section, which provides these details. All the parameters used for the assembly pipeline can be found in the VGP github, from which our pipeline is derived and includes all the scripts and parameters used. The following websites will be added in the Additional File 1.

https://github.com/VGP/vgp-assembly/tree/master/pipeline
For example, for the bionano scaffolding step the config.xml file: https://github.com/VGP/vgp-assembly/blob/master/pipeline/bionano/hybridScaffold_DLE1_config.xml
For salsa we used the default parameters: https://github.com/VGP/vgp-assembly/blob/master/pipeline/salsa/salsa2.2.sh
For 10X scaffolding, you can see the parameters used here: https://github.com/VGP/vgp-assembly/blob/master/pipeline/scaff10x/scaff10x.sh
The falcon unzip parameters can be found here: https://github.com/VGP/vgp-assembly/blob/master/dx_workflows/vgp_falcon_and_unzip_assembly_workflow/dxworkflow.json

The assembly pipeline was run on DNanexus, with the default parameters and the reads filtered using "min_read_length": 500 and "target_coverage": 50.

The remaining software specific parameters are now present in the text. All RepeatMasker analyses are embedded in the text and commands have been added to Additional File 1.

BUSCOs parameters are also specified in the text and commands have been added to Additional File 1.

The MITOS2 server ran the annotation of the mitogenome with the default parameters.

3. The detailed scaffolding step was also missed for the Arima Hi-C data with Salsa 2.2 [18]. How authors deal with the sequence order? This information could help us to understand how the authors addressed the technical issue such as orientation for the inversion regions within the scaffolds.

Thanks for pointing out this matter. The sequence order is not something we considered specifically, but we suggest that these technical issues should not cause any substantial problems for our assembly, given that the contigs we assembled are of exceptionally long lengths and we used two types of scaffolding technology data, with which the types of errors proposed by the reviewer are unlikely to affect our assembly. Specifically, SALSA2 software paper [2] explains how short contigs lead to higher amounts of misoriented contigs within scaffolds, and outperforms its previous version in this regard.

4. The gapless mitochondrial genomes were assembled by PacBio long reads and 10X short reads, and were annotated the by using the MITOS2 web server. The short sequencing reads were typically chosen and used for most mitochondrial genome assembly. Please explain why both the long reads and short reads were chosen during the assembly, or whether this combined strategy presents any advantages compare with traditional method? In addition, in the annotation process for mitogenome, MITOS2 web server was employed, but the descriptions of the procedures could not been found. The details how to reorder and concatenate the annotated genes and regions are appriciate.

The reviewer raises an important point, and we should have been more clear about it in the manuscript. Details regarding the mitogenome assembly process were recently published (Formenti et al. 2021) as part of the broader mitoVGP pipeline, which we have now clarified and cited. The advantage of our combined short-and-long read strategy is that the highly repetitive nature of the mitochondrial control

region (CR) sometimes does not allow for complete error-free assemblies of the mitogenome using short-read data alone. In this specific case there is a small repeat region which is correctly assembled using both long and short reads to obtain the complete mitogenome. We have added the corresponding explanation and citation in the main text.

For the annotation we used MITOS2, a web server that easily annotates genes and regions of any mitochondrial genome. Further details on the procedures can be found in [3], and the corresponding github repository (https://github.com/gavieira/mitos2_wrapper), where you will find the code and specifics of the software, which we did not modify.

5. Please format the references into same style. For example, in reference 19, vs. reference 20. Please revise all "Lemur catta" into italic. Please check and revise according to the policy of GigaScience.

We apologize for this oversight. All references have now been correctly formatted according to GigaScience policy using reference software.

6. Did the author confused the order between Figure 3 and Figure 4?

We apologize for the confusion. We have now reordered the figures during the submission process of the manuscript.

Reviewer #2:

This is a great work conducting genome assembly of this primate species. The assembly would highly benefit from the annotation of the genome (gene annotation) using RNA seq data, however, this seems to be beyond the goals of this manuscript.
Since the focus of the study is on the genome assembly, it would be helpful to conduct Chrimosome Synteny analysis with human genome and other primate species to give a big picture of the differences across the species.
Below, please the comments to this work.

We very much appreciate the reviewer's kind response and positive assessment. The suggestion of a chromosome synteny analysis is an excellent one, which is described below.

Abstract:

Continuous Long Read (CLR) NOT (CLR Reads)? Isn't the word "Read" already included in the abbreviation? Not sure what is the standard abbreviation for this term, and if it really needs mentioning the word "Read".

Thank you for pointing out this oversight. We have adjusted both references from "(CLR reads)" to "(CLR data)". "CLR reads" is a commonly used expression in the field, but we agreed that changing it to "CLR data" makes more sense.

Data Description:

* Any data on the quality of HMW DNA evaluation? Would be good to cite this data in the first paragraph of the Data Description where the authors mention HMW DNA quality control.

We used a PFGE gel (Sage Pippin Pulse) as a HMW quality control measure. We have added the corresponding image as a supplementary figure ("Figure S1: Pulse Field Gel assay (Sage Pippin Pulse) with HMW ladder used for quality control of the ultra-High Molecular Weight DNA (Lemur catta is in well number 1)") and the corresponding text ("uHMW DNA quality was assessed by a Pulsed Field Gel assay and quantified with a Qubit 2 Fluorometer (Figure S1)") in the manuscript as suggested.

* Would be great for the authors to report the results of repeat analysis using Repeat Modeler.

Thank you for this suggestion, which we have given substantial consideration. We decided to run RepeatMasker exclusively for several reasons, but primarily, because there is a high likelihood that a comparable outcome would be produced by RepeatModeler. Additionally, RepeatModeler's results would

lack power for comparison between species, because it depends directly on the quality of the assemblies used. More specifically, running RepeatModeler requires the use of a previously established repeat library in order to classify the repeats present in the focal genome to obtain a specific database of repeats for the genome masking. The standard library in this case would be Dfam, which is also what we used for our RepeatMasker run to classify repeats. We suggest that the well-established primates database provided by RepeatMasker, which is derived from a larger number of genomes, is an appropriate choice for the masking of a lemur genome; thus, we are more confident in our results than we otherwise would be by creating a new database based solely on the present genome. Secondly, RepeatModeler is a well-known and commonly used software and the already complete database it provides will allow for more systematic comparison and analysis by other researchers. Creating a database based on the Lemur catta genome alone could help to find specific repeat patterns within the species, but ultimately, it would still be based on the same previously known library of repeats that RepeatMasker uses to classify them. As such, we think that the computational hurdle of running RepeatModeler would not substantially alter our results.

* Any Synteny analysis compared to other primate species? One of the most useful information from a long-read sequencing (and chromosome-level assembly) is the ability to compare the chromosomal synteny with other primates (or just with humans).

We thank the reviewer for drawing our attention to this issue, and agree that this assembly can be a powerful tool for chromosomal comparison and finding syntenies between Lemur catta and other species. For this purpose, we did a synteny analysis creating a dot plot using Mummer v3.23 software's nucmer -mum option and visualized the results of the synteny between the present assembly of Lemur catta (mLemCat1) and an assembly of Homo sapiens (hg38) using the https://dot.sandbox.bio/ website. We have added this synteny plot as a supplementary figure and the following text to the manuscript:

"The present assembly (mLemCat1) can be useful to create synteny plots between L. catta and others, such as humans (Figure S2), as it has N50 statistics comparable to other high-quality primate genomes recently published (Table S2)."

We added the Figure S2: An overall chromosomal synteny plot between Lemur catta (mLemCat1 assembly) and Homo sapiens (hg38 assembly) in the supplementary material file.

* What is the number of scaffolds that cover 90% of the genome? How different is this number (the number of scaffolds that cover 90% of the genome) compared to the number of chromosomes for this species? Also, what about N95? Would be good to discuss these statistics more clearly to give a clearer picture of the assembly.

The reviewer raises a good point, which we should have been more clear about in the text. We agree that N50/90/95 and L50/90/95 are important statistics to evaluate a genome assembly landscape. In order to keep the text concise, we have adjusted the manuscript to include both N/L50 and N/L95, but include the additional N/L90 values in the supplemental materials, given their similarity to the N/L90 values. The number of scaffolds that cover 90% of the genome is 24, which is 3 more than that found in the hg38 human assembly (L90 = 21). Regarding the L95 and N95 values, we see a similar trend: mLemCat1 L95 = 28 and N95 = 21.9 Mb; hg38 N95 = 24 and N95 = 46.7 Mb. As the Lemur catta genome is about two-thirds the size of the human genome these contiguity values are similar. Additionally, the expected haploid number of chromosomes [4] in Lemur catta is larger, 29 chromosomes expected (27 autosomal + 2 sexual (reference chromosomes)) is larger than the 22 autosomes and 2 sexual human chromosomes. We have added the following parameters in Table S1:

"Lemur catta (mLemCat1): L90 = 24, N90 = 30,322,482 bp ; L95 = 28, N95 = 21,924,082 bp, Span = 2,122,351,751 bp
Human (hg38): L90 = 21, N90 = 58,617,616 bp ; L95 = 24, N95 = 46,709,983 bp, Span = 3,209,286,105 bp"

* What other primate species genomes were recently assembled at "chromosome-level assembly" similar to this study and how the N50 of scaffolds from other recent primate genome assemblies is different (or similar) to N50 scaffold size of this assembly? Would be good to mention in the discussion section. There are a few other recent assemblies of primates in GigaScience (over the last 2 years) using similar methods.

Thank you for this suggestion. As the reviewer rightly mentions, there are other recent primate

assemblies published in GigaScience that are valuable points of comparison. While searching the GigaScience website for published chromosome-level genomes from the past two years, we were able to identify three such assemblies: Ma2, Panubis1.0, and ASM756505v1. mLemcat1 has a slightly smaller scaffold N50 compared to these recently published primate genomes. However, the size of our Lemur catta genome assembly is at least 25% smaller than the other genome assemblies used for this comparison, which explains its proportionally smaller N50 value. We have added the Table S5: Comparison of scaffold N50 and assembly size of the latest primate genomes published in GigaScience to the supplementary materials and the corresponding text in the discussion.

* Repeat analysis would benefit from running 'repeat modeler' in addition to existing analysis.

As we have detailed above, we are confident in our RepeatMasker results and contend that the additional run of Repeat Modeler could lead to additional complications.

1. Sauther ML, Sussman RW, Gould L. The socioecology of the ringtailed lemur: Thirty-five years of research. Evol Anthropol. Wiley; 1999;8:120–32.
2. Ghurye J, Rhie A, Walenz BP, Schmitt A, Selvaraj S, Pop M, et al. Integrating Hi-C links with assembly graphs for chromosome-scale assembly. PLoS Comput Biol. 2019;15:e1007273.
3. Donath A, Jühling F, Al-Arab M, Bernhart SH, Reinhardt F, Stadler PF, et al. Improved annotation of protein-coding genes boundaries in metazoan mitochondrial genomes. Nucleic Acids Res. Oxford Academic; 2019;47:10543–52.
4. Cardone MF, Ventura M, Tempesta S, Rocchi M, Archidiacono N. Analysis of chromosome conservation in Lemur catta studied by chromosome paints and BAC/PAC probes. Chromosoma. 2002;111:348–56.
5. Roodgar M, Babveyh A, Nguyen LH, Zhou W, Sinha R, Lee H, et al. Chromosome-level de novo assembly of the pig-tailed macaque genome using linked-read sequencing and HiC proximity scaffolding. Gigascience. 2020;9.
6. Batra SS, Levy-Sakin M, Robinson J, Guillory J, Durinck S, Vilgalys TP, et al. Accurate assembly of the olive baboon (Papio anubis) genome using long-read and Hi-C data. Gigascience. 2020;9.
7. Wang L, Wu J, Liu X, Di D, Liang Y, Feng Y, et al. A high-quality genome assembly for the endangered golden snub-nosed monkey (Rhinopithecus roxellana). Gigascience. 2019;8.

Close
