# Supplementary material for: A high-quality, long-read genome assembly of the endangered ring-tailed lemur (Lemur catta)
Source: Gigascience. 2022 Apr 1;11:giac026. doi: 10.1093/gigascience/giac026 (PMC8975718; doi:10.1093/gigascience/giac026)
Supplement: giac026_Reviewer_1_Report_Original_Submission — Xiao-Guang Qi -- 11/15/2021 Reviewed [file giac026_reviewer_1_report_original_submission.pdf]

**Reviewer Report**

**Title: Data Note: A high-quality, long-read genome assembly of the endangered ring-tailed lemur (Lemur catta)**

**Version: Original Submission** **Date:** 11/15/2021

**Reviewer name: Xiao-Guang Qi**

**Reviewer Comments to Author:**

The manuscript of "A high-quality, long-read genome assembly of the endangered ring-tailed lemur (Lemur catta)" reports a updated genome assembly for ring-tailed lemur (Lemur catta), a Strepsirrhine primate species. In combination with PacBio continuous long reads (CLR reads), Bionano reads, HiC data, and 10X linked-reads, the contig and scaffold N50 in the newly acquired genome assembly each reached to 10.570 Mbp and 90.982 Mbp. This genome assembly statistic represents 20.41 fold and 421.21 fold increases, respectively, which high quality reference genome could be served as a valuable data resource compared with the previous short-read genome of the species. As the first reported long read assembly for a Lemuriformes, one infraorder within Strepsirrhine, this genomic resource distinguished with previous report which typically focused on higher-primate, especially the apes and old-world monkeys. The release of this genome could potentially facilitate further comparable genomic analysis, help on the understanding of adaptive evolution in primates from Strepsirrhine to Haplorrhini. This updated genome is expected to gain more attention in the research areas of comparative genomics, genetics, conservation and behavior in primates as well as mammals.
The manuscript is well written, technically correct. I suggest accept this paper after minor revision. Some questions belowing may be helpful to improve the manuscript.

1. In the introduction section, beside background of distribution and taxonomy of ring-tailed lemurs, more information will be appreciate including phylogeny position and their biological background such as diet, behavior on so on.
2. During the de novo assembly and subsequent analysis, the authors use several different software packages for their analysis. However, the specific parameter settings for the software used were not given.
3. The detailed scaffolding step was also missed for the Arima Hi-C data with Salsa 2.2 [18]. How authors deal with the sequence order? This information could help us to understand how the authors addressed the technical issue such as orientation for the inversion regions within the scaffolds.
4. The gapless mitochondrial genomes were assembled by PacBio long reads and 10X short reads, and were annotated the by using the MITOS2 web server. The short sequencing reads were typically chosen and used for most mitochondrial genome assembly. Please explain why both the long reads and short reads were chosen during the assembly, or whether this combined strategy presents any advantages compare with traditional method? In addition, in the annotation process for mitogenome, MITOS2 web server was employed, but the descriptions of the procedures could not been found. The details how to reorder and concatenate the annotated genes and regions are appriciate.
5. Please format the references into same style. For example, in reference 19, vs. reference 20. Please

revise all "Lemur catta" into italic. Please check and revise according to the policy of GigaScience.
6. Did the author confused the order between Figure 3 and Figure 4?

**Level of Interest**

Please indicate how interesting you found the manuscript: Choose an item.

**Quality of Written English**

Please indicate the quality of language in the manuscript: Choose an item.

**Declaration of Competing Interests**

Please complete a declaration of competing interests, considering the following questions:

- Have you in the past five years received reimbursements, fees, funding, or salary from an organisation that may in any way gain or lose financially from the publication of this manuscript, either now or in the future?
- Do you hold any stocks or shares in an organisation that may in any way gain or lose financially from the publication of this manuscript, either now or in the future?
- Do you hold or are you currently applying for any patents relating to the content of the manuscript?
- Have you received reimbursements, fees, funding, or salary from an organization that holds or has applied for patents relating to the content of the manuscript?
- Do you have any other financial competing interests?
- Do you have any non-financial competing interests in relation to this paper?

If you can answer no to all of the above, write 'I declare that I have no competing interests' below. If your reply is yes to any, please give details below.

I declare that I have no competing interests

I agree to the open peer review policy of the journal. I understand that my name will be included on my report to the authors and, if the manuscript is accepted for publication, my named report including any attachments I upload will be posted on the website along with the authors' responses. I agree for my report to be made available under an Open Access Creative Commons CC-BY license (http://creativecommons.org/licenses/by/4.0/). I understand that any comments which I do not wish to be included in my named report can be included as confidential comments to the editors, which will not be published.

Choose an item.

To further support our reviewers, we have joined with Publons, where you can gain additional credit to further highlight your hard work (see: https://publons.com/journal/530/gigascience). On publication of this paper, your review will be automatically added to Publons, you can then choose whether or not to claim your Publons credit. I understand this statement.

Yes Choose an item.
