# Supplementary material for: A high-quality, long-read genome assembly of the endangered ring-tailed lemur (Lemur catta)
Source: Gigascience. 2022 Apr 1;11:giac026. doi: 10.1093/gigascience/giac026 (PMC8975718; doi:10.1093/gigascience/giac026)
Supplement: giac026_Reviewer_2_Report_Original_Submission — Morteza Roodgar -- 11/29/2021 Reviewed [file giac026_reviewer_2_report_original_submission.pdf]

Reviewer Report

Title: Data Note: A high-quality, long-read genome assembly of the endangered ring-tailed lemur (Lemur catta)

Version: Original Submission **Date:** 11/29/2021

Reviewer name: Morteza Roodgar

Reviewer Comments to Author:

This is a great work conducting genome assembly of this primate species.
The assembly would highly benefit from the annotation of the genome (gene annotation) using RNA seq data, however, this seems to be beyond the goals of this manuscript.
Since the focus of the study is on the genome assembly, it would be helpful to conduct Chrimosome Synteny analysis with human genome and other primate species to give a big picture of the differences across the species.
Below, please the comments to this work.
Abstract:
Continuous Long Read (CLR) NOT (CLR Reads)? Isn't the word "Read" already included in the abbreviation? Not sure what is the standard abbreviation for this term, and if it really needs mentioning the word "Read".
Data Description:
* Any data on the quality of HMW DNA evaluation? Would be good to cite this data in the first paragraph of the Data Description where the authors mention HMW DNA quality control.
* Would be great for the authors to report the results of repeat analysis using Repeat Modeler.
* Any Synteny analysis compared to other primate species? One of the most useful information from a long-read sequencing (and chromosome-level assembly) is the ability to compare the chromosomal synteny with other primates (or just with humans).
* What is the number of scaffolds that cover 90% of the genome? How different is this number (the number of scaffolds that cover 90% of the genome) compared to the number of chromosomes for this species? Also, what about N95? Would be good to discuss these statistics more clearly to give a clearer picture of the assembly.
* What other primate species genomes were recently assembled at "chromosome-level assembly" similar to this study and how the N50 of scaffolds from other recent primate genome assemblies is different (or similar) to N50 scaffold size of this assembly? Would be good to mention in the discussion section. There are a few other recent assemblies of primates in GigaScience (over the last 2 years) using similar methods.
* Repeat analysis would benefit from running 'repeat modeler' in addition to exisiting analysis.

Level of Interest

Please indicate how interesting you found the manuscript: Choose an item.

Quality of Written English

Please indicate the quality of language in the manuscript: Choose an item.

Declaration of Competing Interests

Please complete a declaration of competing interests, considering the following questions:

- Have you in the past five years received reimbursements, fees, funding, or salary from an organisation that may in any way gain or lose financially from the publication of this manuscript, either now or in the future?
- Do you hold any stocks or shares in an organisation that may in any way gain or lose financially from the publication of this manuscript, either now or in the future?
- Do you hold or are you currently applying for any patents relating to the content of the manuscript?
- Have you received reimbursements, fees, funding, or salary from an organization that holds or has applied for patents relating to the content of the manuscript?
- Do you have any other financial competing interests?
- Do you have any non-financial competing interests in relation to this paper?

If you can answer no to all of the above, write 'I declare that I have no competing interests' below. If your reply is yes to any, please give details below.

I declare that I have no competing interests

I agree to the open peer review policy of the journal. I understand that my name will be included on my report to the authors and, if the manuscript is accepted for publication, my named report including any attachments I upload will be posted on the website along with the authors' responses. I agree for my report to be made available under an Open Access Creative Commons CC-BY license (http://creativecommons.org/licenses/by/4.0/). I understand that any comments which I do not wish to be included in my named report can be included as confidential comments to the editors, which will not be published.

Choose an item.

To further support our reviewers, we have joined with Publons, where you can gain additional credit to further highlight your hard work (see: https://publons.com/journal/530/gigascience). On publication of this paper, your review will be automatically added to Publons, you can then choose whether or not to claim your Publons credit. I understand this statement.

Yes Choose an item.
