# Supplementary material for: A high-quality, long-read genome assembly of the endangered ring-tailed lemur (Lemur catta)
Source: Gigascience. 2022 Apr 1;11:giac026. doi: 10.1093/gigascience/giac026 (PMC8975718; doi:10.1093/gigascience/giac026)
Supplement: giac026_Reviewer_2_Report_Revision_1 — Morteza Roodgar -- 2/8/2022 Reviewed [file giac026_reviewer_2_report_revision_1.pdf]

Reviewer Report

Title: Data Note: A high-quality, long-read genome assembly of the endangered ring-tailed lemur (Lemur catta)

Version: Revision 1 **Date:** 2/8/2022

Reviewer name: Morteza Roodgar

Reviewer Comments to Author:

I think the authors addressed almost all of the issues raised about the paper and they did a great job in responding to the comments. However, with regards to one comment on repeat analysis, I suggested author to use Repeat Modeler (in addition to Repeat Masker) to verify the results of repeat analysis that was done using Repeat Masker. I agree that the results would be similar (very likely) but a "speculation" by authors that the result of Repeat Modeler would be the same as Repeat Masker is not a great answer to this comment. I think it would be better to run Repeat Modeler and actually show the results are similar without any speculation. Even if the results of Repeat Modeller and Repeat Masker will not be similar, the authors can discuss this in the discussion and it would not be a weak point of the study. Having said that, this is not a point that stops the paper from publishing and I recommend the acceptance of the paper (unless the editors would prefer to address this comment before publishing).

Level of Interest

Please indicate how interesting you found the manuscript: Choose an item.

Quality of Written English

Please indicate the quality of language in the manuscript: Choose an item.

Declaration of Competing Interests

Please complete a declaration of competing interests, considering the following questions:

- Have you in the past five years received reimbursements, fees, funding, or salary from an organisation that may in any way gain or lose financially from the publication of this manuscript, either now or in the future?
- Do you hold any stocks or shares in an organisation that may in any way gain or lose financially from the publication of this manuscript, either now or in the future?
- Do you hold or are you currently applying for any patents relating to the content of the manuscript?
- Have you received reimbursements, fees, funding, or salary from an organization that holds or has applied for patents relating to the content of the manuscript?
- Do you have any other financial competing interests?

- Do you have any non-financial competing interests in relation to this paper?

If you can answer no to all of the above, write 'I declare that I have no competing interests' below. If your reply is yes to any, please give details below.

I declare that I have no competing interests

I agree to the open peer review policy of the journal. I understand that my name will be included on my report to the authors and, if the manuscript is accepted for publication, my named report including any attachments I upload will be posted on the website along with the authors' responses. I agree for my report to be made available under an Open Access Creative Commons CC-BY license (http://creativecommons.org/licenses/by/4.0/). I understand that any comments which I do not wish to be included in my named report can be included as confidential comments to the editors, which will not be published.

Choose an item.

To further support our reviewers, we have joined with Publons, where you can gain additional credit to further highlight your hard work (see: https://publons.com/journal/530/gigascience). On publication of this paper, your review will be automatically added to Publons, you can then choose whether or not to claim your Publons credit. I understand this statement.

Yes Choose an item.
